# Absolute Risk of Adverse Obstetric Outcomes Among Twin Pregnancies After In Vitro Fertilization by Maternal Age

**DOI:** 10.1001/jamanetworkopen.2021.23634

**Published:** 2021-09-10

**Authors:** Yuanyuan Wang, Huifeng Shi, Lian Chen, Danni Zheng, Xiaoyu Long, Yunjun Zhang, Haibo Wang, Ying Shi, Yangyu Zhao, Yuan Wei, Jie Qiao

**Affiliations:** 1 Center for Reproductive Medicine, Peking University Third Hospital, Beijing, China; 2 Department of Obstetrics and Gynecology, Peking University Third Hospital, Beijing, China; 3 National Clinical Research Center for Obstetrical and Gynecology, Peking University Third Hospital, Beijing, China; 4 Key Laboratory of Assisted Reproduction, Peking University, Ministry of Education, Beijing, China; 5 National Center for Healthcare Quality Management in Obstetrics, Beijing, China; 6 Department of Biostatistics, Peking University School of Public Health, Beijing, China; 7 Clinical Trial Unit, First Affiliated Hospital of Sun Yat-Sen University, Guangzhou, China; 8 Centre for Data Science in Health and Medicine, Peking University, Beijing, China; 9 China Standard Medical Information Research Center, Shenzhen, China

## Abstract

**Importance:**

Twin pregnancy is a common occurrence in pregnancies conceived with in vitro fertilization (IVF), but the absolute risk of adverse obstetric outcomes stratified by IVF, twin or singleton pregnancy, and maternal age are unknown.

**Objective:**

To estimate the absolute risk of adverse obstetric outcomes at each maternal age among twin pregnancies conceived with IVF.

**Design, Setting, and Participants:**

This retrospective cohort study included pregnant women with infants born from January 1, 2013, to December 31, 2018, based on the Hospital Quality Monitoring System in China. Data were analyzed from September 1, 2020, to June 30, 2021.

**Exposures:**

Twin pregnancy with IVF (IVF-T), singleton pregnancy with IVF (IVF-S), twin pregnancy with non-IVF (nIVF-T), and singleton pregnancy with non-IVF (nIVF-S).

**Main Outcomes and Measures:**

Sixteen obstetric outcomes, including 10 maternal complications (gestational hypertension, eclampsia and preeclampsia, gestational diabetes, placenta previa, placental abruption, placenta accreta, preterm birth, dystocia, cesarean delivery, and postpartum hemorrhage) and 6 neonatal complications (fetal growth restriction, low birth weight, very low birth weight, macrosomia, malformation, and stillbirth).

**Results:**

Among 16 879 728 pregnant women aged 20 to 49 years (mean [SD] age, 29.2 [4.7] years), the twin-pregnancy rates were 32.1% in the IVF group and 1.5% in the non-IVF group (relative risk, 20.8; 95% CI, 20.6-20.9). The most common adverse obstetric outcomes after pregnancy conceived with IVF were cesarean delivery (88.8%), low birth weight (43.8%), preterm birth (39.6%), gestational diabetes (20.5%), gestational hypertension and preeclampsia and eclampsia (17.5%), dystocia (16.8%), and postpartum hemorrhage (11.9%). The absolute risk of most adverse obstetric outcomes in each subgroup presented in 2 dominant patterns: Pattern A indicated the absolute risk ranging from IVF-T to nIVF-T to IVF-S to nIVF-S, and pattern B indicated the absolute risk ranging from IVF-T to IVF-S to nIVF-T to nIVF-S. Both patterns showed an elevated obstetric risk with increasing maternal age in each subgroup.

**Conclusions and Relevance:**

In this cohort study, twin pregnancy, IVF, and advanced maternal age were independently associated with adverse obstetric outcomes. Given these findings, promotion of the elective single embryo transfer strategy is needed to reduce multiple pregnancies following IVF technologies. Unnecessary cesarean delivery shouldh be avoided in all pregnant women.

## Key Points

**Question:**

What is the absolute risk of adverse obstetric outcomes stratified by in vitro fertilization (IVF), twin or singleton pregnancy, and maternal age?

**Findings:**

In this cohort study of 16 879 728 pregnant women, the twin pregnancy rate was 32.1% among those who conceived via IVF. Twin pregnancies conceived via IVF had higher absolute obstetric risks in each maternal age compared with IVF-conceived singleton pregnancies or non–IVF-conceived twin pregnancies.

**Meaning:**

These findings suggest that twin pregnancy, IVF, and advanced maternal age are independently associated with adverse obstetric outcomes, and their coexistence may lead to the aggravation of obstetric risk.

## Teaser

This cohort study estimates the absolute risk of maternal and neonatal complications among women who gave birth in China during a 6-year period stratified by in vitro fertilization, twin or singleton pregnancy, and maternal age.

## Introduction

In vitro fertilization (IVF) technologies have developed and spread globally during the past 4 decades since the first IVF-conceived infant was born in 1978.^1^ Currently, IVF technologies mainly include IVF and embryo transfer, intracytoplasmic sperm injection, frozen embryo transfer, and preimplantation genetic testing, which have been widely applied among couples with infertility or monogenic diseases or with the intention of fertility preservation.^1,2^ The updated global estimated number of IVFs per year was approximately 2.8 million initiated cycles and 0.9 million infants in 2012.^3^ In China, there were 906 840 IVF cycles and 289 836 IVF infants in 2016,^4^ accounting for 1.6% of the total 17.86 million births in the whole country in the same year.^5^

Twin pregnancy is a common occurrence in pregnancies conceived with IVF because multiple embryo transfer is commonly regarded as an effective strategy to improve the likelihood of a successful pregnancy.^6^ Globally, the twin delivery rates after IVF were 18.0% among fresh nondonor IVF embryo transfer and intracytoplasmic sperm injection cycles and 11.1% among frozen embryo transfer nondonor cycles in 2012, varying to some extent in different countries and regions.^3^ In 2016, such twin delivery rates in Europe were 14.9% and 11.9%, respectively.^7^ The rates in the US were 18.8% and 13.7%, respectively,^8^ whereas twin delivery rates in China were 27.9% among fresh IVF embryo transfer cycles, 27.2% among fresh intracytoplasmic sperm injection cycles, and 24.2% among frozen embryo transfer cycles (egg and embryo donation are rarely performed in China).^4^ Compared with singleton pregnancies, twin pregnancies are significantly linked with increased perinatal morbidity and mortality, including maternal near-death events, preterm birth, low birth weight, cesarean delivery, admission to the neonatal intensive care unit, stillbirth, and perinatal mortality.^9,10,11,12^ These adverse obstetric outcomes are more likely to occur among pregnant women who underwent IVF conception than among those who conceived naturally.^13,14,15,16^ Therefore, the risk of adverse obstetric outcomes among twin pregnancies after IVF may confoundingly originate from both twin pregnancy and IVF. In addition, maternal age is a critical independent factor for obstetric outcomes, whether after IVF or natural conception,^17,18,19^ and presents nonlinear associations with many outcomes.^17^ However, the risks of obstetric outcomes stratified by IVF, twin or singleton pregnancy, and maternal age are unknown. This study aimed to estimate the absolute risk of obstetric outcomes stratified by IVF or non-IVF conception and twin or singleton pregnancy at each maternal age to accurately evaluate the obstetric risks among twin pregnancies after IVF and then to develop management strategies in both IVF procedures and obstetric health care to ensure the health of mothers and infants.

## Methods

The Science Research Ethics Committee of the Peking University Third Hospital Medical, Beijing, China, approved this retrospective cohort study and determined that it was exempt from human participant research review and that informed consent could be waived because this was a secondary data analysis with no personally identifiable information. Our study conforms to the Strengthening the Reporting of Observational Studies in Epidemiology (STROBE) reporting guidelines for cohort studies.

### Study Design and Population

**Figure 1.**
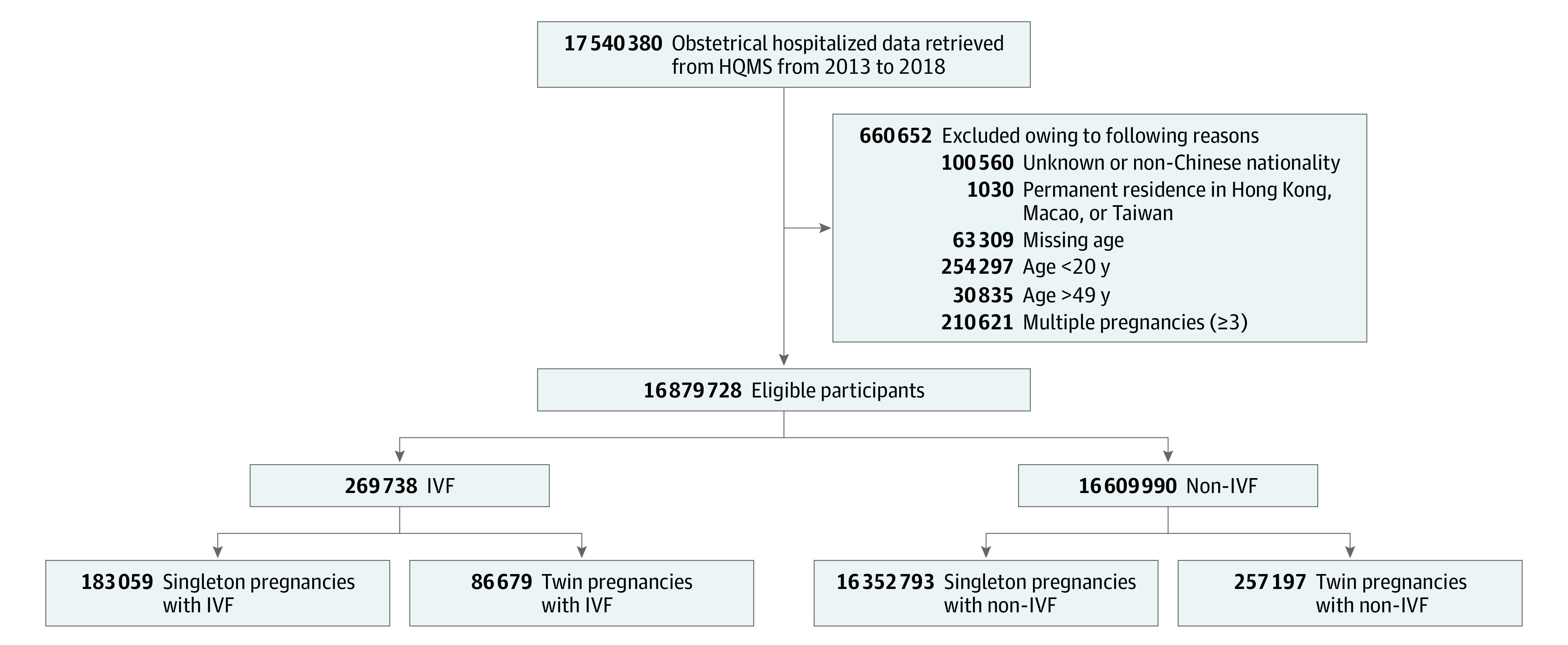
Flowchart of Data According to Eligibility for Inclusion in This Study HQMS indicates Hospital Quality Monitoring System; IVF, in vitro fertilization.

This study was a retrospective analysis based on the Hospital Quality Monitoring System (HQMS), established by the National Health Commission of China. Since 2013, the HQMS has automatically collected standardized electronic hospitalized discharge records from class 3 hospitals in China, which are also called tertiary hospitals (all hospitals in China are certificated by the government as class 1, 2, or 3, with class 3 being the highest grade, according to the scale of facilities, number of patients, level of technology, quality of care, and other standards^20^). In the HQMS database, all diagnoses of disorders and diseases were coded using the *International Statistical Classification of Diseases and Related Health Problems, Tenth Revision* (*ICD-10*)^21^; all operating procedures were coded by using the *ICD-10* and the *International Classification of Diseases, 9th Revision, Clinical Modification* (*ICD-9-CM*).^22^ All *ICD-10* and *ICD-9-CM* codes of variables used in this study are summarized in eTable 1 in the Supplement. The number of class 3 hospitals in HQMS increased from 823 to 1853 from 2013 to 2018; the proportion of class 3 hospitals relative to all hospitals in China increased from 46.1% to 72.7%.^23^ In this study, we retrieved all obstetrical hospitalized data from the HQMS according to the inclusion criteria: pregnant women with live birth at a gestational age of at least 28 weeks or stillbirth at a gestational age of at least 20 weeks. A total of 17 540 380 pregnant women were drawn from the HQMS database from January 1, 2013, to December 31, 2018. The exclusion criteria were as follows: (1) nationality unknown or not Chinese; (2) permanent residence in Hong Kong, Macao, and Taiwan; (3) maternal age missing, younger than 20 years, or older than 49 years; and (4) pregnancy with triplets or more. Finally, 16 879 728 pregnant women were eligible for inclusion in this study, including 269 738 women who conceived with IVF and 16 609 990 women who conceived without any IVF technology (Figure 1).

### Variable Definitions

The variables used in this study can be divided into 4 categories. Categories included maternal group, sociodemographic characteristics, maternal chronic diseases, and obstetric outcomes.

#### Maternal Group

In vitro fertilization was defined as pregnancy conceived with any technology of IVF. In vitro fertilization or non-IVF was identified according to the *ICD-9-CM* or *ICD-10* codes of IVF procedures. Singleton pregnancy or twin pregnancy was also identified by the related *ICD-9-CM* or *ICD-10* codes. The study population was then divided into 4 subgroups: singleton pregnancy with IVF (IVF-S), twin pregnancy with IVF (IVF-T), singleton pregnancy with non-IVF (nIVF-S), and twin pregnancy with non-IVF (nIVF-T).

#### Sociodemographic Characteristics of the Pregnant Women

The geographic region was defined as the hospital’s location. The eastern region includes 11 provinces: Beijing, Tianjin, Hebei, Liaoning, Shanghai, Jiangsu, Zhejiang, Fujian, Shandong, Guangdong, and Hainan. The central region includes 8 provinces: Shanxi, Jilin, Heilongjiang, Anhui, Jiangxi, Henan, Hubei, and Hunan. The western region includes 12 provinces: Sichuan, Chongqing, Guizhou, Yunnan, Tibet, Shaanxi, Gansu, Qinghai, Ningxia, Xinjiang, Guangxi, and Inner Mongolia. Year was defined as the discharge date of the pregnant woman. Maternal age was defined as the pregnant woman’s age at delivery (including either live birth or stillbirth). Ethnicity was defined as the ethnic group (Han or minority) to which the pregnant woman belonged.

#### Maternal Chronic Diseases

Maternal chronic diseases were defined as chronic diseases that the pregnant woman had before pregnancy, including chronic hypertension, diabetes, thyroid diseases, anemia, circulatory diseases, and other diseases (coagulation disorders, kidney diseases, diseases of connective tissues, diseases of the respiratory system, and diseases of the digestive system). All variables in this category were identified according to the *ICD-9-CM* or *ICD-10* codes.

#### Obstetric Outcomes

Maternal outcomes were defined as maternal complications that developed during pregnancy, including gestational hypertension, eclampsia and preeclampsia, gestational diabetes, placenta previa, placental abruption, placenta accreta, preterm birth (gestational age at birth, 28-36 weeks), dystocia, cesarean delivery, and postpartum hemorrhage (bleeding volume ≥500 mL after vaginal delivery or ≥1000 mL after cesarean delivery). Neonatal outcomes were defined as neonatal complications that developed before or after birth until discharge, including fetal growth restriction (an estimated fetal weight during ultrasonographic screening that is less than the 10th percentile for gestational age), low birth weight (<2500 g), very low birth weight (<1500 g), macrosomia (birth weight >4000 g), malformation (congenital malformations, deformations, and chromosomal abnormalities), and stillbirth (the death or loss of an infant before or during delivery at 20 weeks of gestational age or later). Among these variables, low birth weight and very low birth weight were identified according to birth weights reported in the records, and other variables were identified according to the related *ICD-9-CM* or *ICD-10* codes.

### Statistical Analysis

Continuous variables are described as means (SD), and comparisons in different groups were performed using the 2-tailed *t* test. Categorical variables are described as counts with percentages, and comparisons in different groups were performed using the χ^2^ test with relative risk (RR) and 95% CI. Poisson regression models using restricted cubic splines of maternal age were performed to examine the risk of each obstetric outcome by each maternal age in each subgroup. The level of significance was defined as *P* = .05, and all hypothesis tests were 2 sided. All analyses were conducted using SAS, version 9.0 software (SAS Institute, Inc).^24^ Data were analyzed from September 1, 2020, to June 30, 2021.

In total, 16 obstetric outcomes were examined in this study, including 10 maternal complications and 6 neonatal complications. For each outcome, we performed 3 Poisson regression models: (1) using only singleton pregnancy data to examine the adjusted RR (aRR) and 95% CI of IVF-S vs nIVF-S (model 1), (2) using only twin pregnancy data to examine the aRR and 95% CI of IVF-T vs nIVF-T (model 2), and (3) using both singleton pregnancy and twin pregnancy data to examine the aRR and 95% CI of IVF vs non-IVF (model 3). Geographic region, maternal age, year, ethnicity, and maternal chronic diseases were adjusted for in all models, and singleton or twin pregnancy was additionally adjusted for in model 3. The interaction effect between IVF and twin pregnancy was tested by including an interaction term in model 3. Maternal age was modeled using restricted cubic splines to allow a nonlinear association with each outcome, and the number of knots placed at the default percentiles was determined according to the principle of minimized Akaike information criterion in the adjusted model.^25,26^ In model 3, we computed estimated absolute risks (probabilities and 95% CIs) in each subgroup and then presented them graphically to visually illustrate the risk of each obstetric outcome by each maternal age.

### Sensitivity Analyses

The number of sampling hospitals in the HQMS increased every year but varied largely from 2013 to 2015 (823 hospitals in 2013, 907 hospitals in 2014, and 920 hospitals in 2015) and from 2016 to 2018 (1810 hospitals in 2016, 1835 hospitals in 2017, and 1853 hospitals in 2018). Sensitivity analyses were performed by using multivariable adjusted analyses stratified by calendar year (before or after 2016).

## Results

### Characteristics of the Study Population

Among a total of 16 879 728 pregnant women aged 20 to 49 years analyzed in this study (mean [SD] age, 29.2 [4.7] years), 269 738 (1.6%) conceived with IVF, and 16 609 990 (98.4%) conceived with non-IVF. The twin pregnancy rate was 32.1% (86 679 women) in the IVF group and 1.5% (257 197 women) in the non-IVF group (RR, 20.8; 95% CI, 20.6-20.9) (Figure 1). From 2013 to 2018, the proportion of all pregnancies conceived with IVF increased from 1.1% to 2.2%, and that of twin pregnancies conceived with IVF increased from 0.4% to 0.7%. The study population of subgroups by year, province, and maternal age is shown in eTables 2 to 4 in the Supplement.

The characteristics of pregnant women and results compared between groups are summarized in Table 1. Compared with the non-IVF group, the IVF group had a higher proportion of pregnant women 35 years or older (28.8% vs 13.7%) and a higher proportion with chronic hypertension (1.5% vs 0.8%), diabetes (1.9% vs 0.9%), thyroid diseases (7.3% vs 3.8%), anemia (18.4% vs 15.8%), circulatory diseases (1.7% vs 1.0%), and other diseases (8.1% vs 4.5%) (*P* < .001 for all).

**Table 1.** Characteristics of Study Population^a^

Characteristic	Total (N = 16 879 728)	IVF pregnancy group			Non-IVF pregnancy group			*P* value		
		Singleton (n = 183 059)	Twin (n = 86 679)	All (n = 269 738)	Singleton (n = 16 352 793)	Twin (n = 257 197)	All (n = 16 609 990)	IVF-S vs nIVF-S	IVF-T vs nIVF-T	All IVF vs non-IVF
Geographic region										
Eastern	8 642 035 (51.2)	101 253 (55.3)	47 925 (55.3)	149 178 (55.3)	8 361 388 (51.1)	131 469 (51.1)	8 492 857 (51.1)	<.001	<.001	<.001
Central	3 932 114 (23.3)	43 965 (24.0)	20 684 (23.9)	64 649 (24.0)	3 808 910 (23.3)	58 555 (22.8)	3 867 465 (23.3)	<.001	<.001	<.001
Western	4 305 579 (25.5)	37 841 (20.7)	18 070 (20.8)	55 911 (20.7)	4 182 495 (25.6)	67 173 (26.1)	4 249 668 (25.6)	<.001	<.001	<.001
Year										
2013	769 987 (4.6)	5477 (3.0)	2855 (3.3)	8332 (3.1)	746 520 (4.6)	15 135 (5.9)	761 655 (4.6)	<.001	<.001	<.001
2014	1 471 619 (8.7)	9791 (5.3)	5438 (6.3)	15 229 (5.6)	1 428 376 (8.7)	28 014 (10.9)	1 456 390 (8.8)	<.001	<.001	<.001
2015	1 360 813 (8.1)	13 383 (7.3)	7546 (8.7)	20 929 (7.8)	1 314 390 (8.0)	25 494 (9.9)	1 339 884 (8.1)	<.001	<.001	<.001
2016	4 454 176 (26.4)	41 992 (22.9)	19 341 (22.3)	61 333 (22.7)	4 328 514 (26.5)	64 329 (25.0)	4 392 843 (26.4)	<.001	<.001	<.001
2017	4 538 105 (26.9)	47 874 (26.2)	22 509 (26.0)	70 383 (26.1)	4 403 157 (26.9)	64 565 (25.1)	4 467 722 (26.9)	<.001	<.001	<.001
2018	4 285 028 (25.4)	64 542 (35.3)	28 990 (33.4)	93 532 (34.7)	4 131 836 (25.3)	59 660 (23.2)	4 191 496 (25.2)	<.001	<.001	<.001
Maternal age, y										
Mean (SD)	29.2 (4.7)	32.5 (4.5)	31.5 (4.1)	32.2 (4.4)	29.2 (4.6)	29.5 (4.7)	29.2 (4.6)	<.001	<.001	<.001
20-24	2 458 076 (14.6)	4859 (2.7)	2417 (2.8)	7276 (2.7)	2 414 955 (14.8)	35 845 (13.9)	2 450 800 (14.8)	<.001	<.001	<.001
25-29	7 314 846 (43.3)	43 033 (23.5)	26 045 (30.0)	69 078 (25.6)	7 141 950 (43.7)	103 818 (40.4)	7 245 768 (43.6)	<.001	<.001	<.001
30-34	4 759 856 (28.2)	76 727 (41.9)	38 813 (44.8)	115 540 (42.8)	4 565 193 (27.9)	79 123 (30.8)	4 644 316 (28.0)	<.001	<.001	<.001
35-39	1 906 461 (11.3)	46 330 (25.3)	16 546 (19.1)	62 876 (23.3)	1 811 630 (11.1)	31 955 (12.4)	1 843 585 (11.1)	<.001	<.001	<.001
40-44	411 245 (2.4)	10 990 (6.0)	2340 (2.7)	13 330 (4.9)	392 006 (2.4)	5909 (2.3)	397 915 (2.4)	<.001	<.001	<.001
45-49	29 244 (0.2)	1120 (0.6)	518 (0.6)	1638 (0.6)	27 059 (0.2)	547 (0.2)	27 606 (0.2)	<.001	<.001	<.001
Ethnicity										
Minority	1 547 989 (9.2)	16 366 (8.9)	7959 (9.2)	24 325 (9.0)	1 498 699 (9.2)	24 965 (9.7)	1 523 664 (9.2)	<.001	<.001	<.001
Han	15 164 669 (89.8)	165 736 (90.5)	78 174 (90.2)	243 910 (90.4)	14 690 758 (89.8)	230 001 (89.4)	14 920 759 (89.8)	<.001	<.001	<.001
Unknown	167 070 (1.0)	957 (0.5)	546 (0.6)	1503 (0.6)	163 336 (1.0)	2231 (0.9)	165 567 (1.0)	<.001	<.001	<.001
Maternal chronic diseases										
Chronic hypertension	138 116 (0.8)	2713 (1.5)	1212 (1.4)	3925 (1.5)	130 338 (0.8)	3853 (1.5)	134 191 (0.8)	<.001	.03	<.001
Diabetes	162 724 (1.0)	3822 (2.1)	1328 (1.5)	5150 (1.9)	154 378 (0.9)	3196 (1.2)	157 574 (0.9)	<.001	<.001	<.001
Thyroid diseases	659 160 (3.9)	13 441 (7.3)	6325 (7.3)	19 766 (7.3)	626 652 (3.8)	12 742 (5.0)	639 394 (3.8)	<.001	<.001	<.001
Anemia	2 673 747 (15.8)	29 171 (15.9)	20 373 (23.5)	49 544 (18.4)	2 560 513 (15.7)	63 690 (24.8)	2 624 203 (15.8)	.001	<.001	<.001
Circulatory diseases	173 609 (1.0)	2665 (1.5)	1972 (2.3)	4637 (1.7)	164 339 (1.0)	4633 (1.8)	168 972 (1.0)	<.001	<.001	<.001
Other diseases^b^	770 041 (4.6)	11 226 (6.1)	10 650 (12.3)	21 876 (8.1)	723 601 (4.4)	24 564 (9.6)	748 165 (4.5)	<.001	<.001	<.001

Abbreviations: IVF, in vitro fertilization; IVF-S, IVF singleton pregnancy; IVF-T, IVF twin pregnancy; nIVF, non-IVF.

^a^ Unless otherwise indicated, data are expressed as number (percentage) of women. Percentages have been rounded and may not total 100.

^b^ Includes coagulation disorders, kidney diseases, diseases of connective tissues, diseases of the respiratory system, and diseases of the digestive system.

### Maternal and Neonatal Outcomes

Table 2 and eTable 5 in the Supplement show the comparisons of obstetric outcomes between the different groups. For 10 categories of maternal outcomes observed in this study, the IVF group had a higher risk than the non-IVF group in gestational hypertension (aRR, 1.37; 95% CI, 1.34-1.40), eclampsia and preeclampsia (aRR, 1.26; 95% CI, 1.24-1.28), gestational diabetes (aRR, 1.45; 95% CI, 1.44-1.47), placenta previa (aRR, 1.82; 95% CI, 1.78-1.85), placental abruption (aRR, 1.11; 95% CI, 1.07-1.15), placenta accreta (aRR, 1.85; 95% CI, 1.81-1.89), preterm birth (aRR, 1.18; 95% CI, 1.16-1.19), dystocia (aRR, 1.33; 95% CI, 1.31-1.34), cesarean delivery (aRR, 1.21; 95% CI, 1.20-1.22), and postpartum hemorrhage (aRR, 1.63; 95% CI, 1.61-1.66). The models showed significant interaction between IVF and twin pregnancy for maternal outcomes except for dystocia (eTable 6 in the Supplement). Further analyses of twin pregnancies showed that the IVF-T group had a higher risk of 9 outcomes than the nIVF-T group, but there was no difference in placental abruption (aOR, 1.03; 95% CI, 0.96-1.10). Notably, the rate of cesarean delivery was fairly high in each subgroup: 88.8% in the IVF-T group, 80.3% in the nIVF-T group, 66.5% in the IVF-S group, and 43.6% in the nIVF-S group. The most common maternal complications following twin pregnancy conceived with IVF were cesarean delivery (88.8%), preterm birth (39.6%), gestational diabetes (20.5%), gestational hypertension and preeclampsia and eclampsia (17.5%), dystocia (16.8%), and postpartum hemorrhage (11.9%).

**Table 2.** Maternal and Neonatal Outcomes in Subgroups^a^

Outcome	Total (N = 16 879 728)	Singleton pregnancy			Twin pregnancy			All pregnancy		
		Non-IVF (n = 16 352 793)	IVF (n = 183 059)	aRR (95% CI)^b^	Non-IVF (n = 257 197)	IVF (n = 86 679)	aRR (95% CI)^c^	Non-IVF (n = 16 609 990)	IVF (n = 269 738)	aRR (95% CI)^d^
**Maternal outcomes**										
Gestational hypertension	289 584 (1.7)	270 174 (1.7)	5962 (3.3)	1.55 (1.51-1.59)^e^	9451 (3.7)	3997 (4.6)	1.14 (1.10-1.19)^e^	279 625 (1.7)	9959 (3.7)	1.37 (1.34-1.40)^e^
Eclampsia and preeclampsia	515 770 (3.1)	465 660 (2.8)	10 284 (5.6)	1.52 (1.49-1.55)^e^	28 692 (11.2)	11 134 (12.8)	1.12 (1.09-1.14)^e^	494 352 (3.0)	21 418 (7.9)	1.26 (1.24-1.28)^e^
Gestational diabetes	1 764 999 (10.5)	1 677 918 (10.3)	38 571 (21.1)	1.48 (1.47-1.50)^e^	30 755 (12.0)	17 755 (20.5)	1.39 (1.36-1.42)^e^	1 708 673 (10.3)	56 326 (20.9)	1.45 (1.44-1.47)^e^
Placenta previa	337 880 (2.0)	319 908 (2.0)	8865 (4.8)	1.87 (1.83-1.91)^e^	5596 (2.2)	3511 (4.1)	1.62 (1.55-1.69)^e^	325 504 (2.0)	12 376 (4.6)	1.82 (1.78-1.85)^e^
Placental abruption	131 759 (0.8)	125 574 (0.8)	1853 (1.0)	1.16 (1.11-1.21)^e^	3181 (1.2)	1151 (1.3)	1.03 (0.96-1.10)	128 755 (0.8)	3004 (1.1)	1.11 (1.07-1.15)^e^
Placenta accreta	338 471 (2.0)	318 321 (1.9)	8554 (4.7)	2.00 (1.96-2.04)^e^	7495 (2.9)	4101 (4.7)	1.46 (1.40-1.52)^e^	325 816 (2.0)	12 655 (4.7)	1.85 (1.81-1.89)^e^
Preterm birth	934 841 (5.5)	792 997 (4.8)	15 066 (8.2)	1.48 (1.46-1.51)^e^	92 417 (35.9)	34 361 (39.6)	1.08 (1.07-1.10)^e^	885 414 (5.3)	49 427 (18.3)	1.18 (1.16-1.19)^e^
Dystocia	1 464 262 (8.7)	1 395 802 (8.5)	20 630 (11.3)	1.34 (1.32-1.36)^e^	33 246 (12.9)	14 584 (16.8)	1.22 (1.20-1.25)	1 429 048 (8.6)	35 214 (13.1)	1.33 (1.31-1.34)^e^
Cesarean section	7 533 321 (44.6)	7 127 990 (43.6)	121 742 (66.5)	1.32 (1.31-1.33)^e^	206 624 (80.3)	76 965 (88.8)	1.08 (1.07-1.09)^e^	7 334 614 (44.2)	198 707 (73.7)	1.21 (1.20-1.22)^e^
Postpartum hemorrhage	695 031 (4.1)	649 616 (4.0)	14 403 (7.9)	1.77 (1.74-1.80)^e^	20 697 (8.0)	10 315 (11.9)	1.43 (1.40-1.47)^e^	670 313 (4.0)	24 718 (9.2)	1.63 (1.61-1.66)^e^
**Neonatal outcomes**										
FGR	152 865 (0.9)	132 517 (0.8)	2205 (1.2)	1.36 (1.30-1.42)^e^	13 566 (5.3)	4577 (5.3)	0.96 (0.93-1.00)^f^	146 083 (0.9)	6782 (2.5)	1.09 (1.06-1.12)^e^
Birth weight^g^										
Low	859 196 (5.7)	721 709 (5.0)	11 688 (7.1)	1.35 (1.32-1.37)^e^	93 477 (43.3)	32 322 (43.8)	1.03 (1.01-1.04)^e^	815 186 (5.5)	44 010 (18.5)	1.10 (1.09-1.11)^e^
Very low	142 507 (1.0)	126 602 (0.9)	2285 (1.4)	1.42 (1.36-1.48)^e^	10 128 (4.7)	3492 (4.7)	1.07 (1.03-1.11)^e^	136 730 (0.9)	5777 (2.4)	1.14 (1.11-1.17)^e^
Macrosomia	880 182 (5.2)	868 381 (5.3)	10 801 (5.9)	0.99 (0.97-1.01)	894 (0.3)	106 (0.1)	0.35 (0.29-0.43)^f^	869 275 (5.2)	10 907 (4.0)	0.98 (0.96-1.00)^f^
Malformation	186 322 (1.1)	170 917 (1.0)	3330 (1.8)	1.53 (1.48-1.58)^e^	9544 (3.7)	2531 (2.9)	0.74 (0.70-0.77)^f^	180 461 (1.1)	5861 (2.2)	1.09 (1.06-1.13)^e^
Stillbirth	73 205 (0.4)	61 273 (0.4)	347 (0.2)	0.46 (0.42-0.51)^f^	9290 (3.6)	2295 (2.6)	0.75 (0.72-0.79)^f^	70 563 (0.4)	2642 (1.0)	0.68 (0.65-0.71)^f^

Abbreviations: aRR, adjusted relative risk; FGR, fetal growth restriction; IVF, in vitro fertilization.

^a^ Unless otherwise specified, data are presented as No. (%).

^b^ The results of model 1, using only singleton pregnancy data, with adjusting for geographic region, maternal age, year, ethnicity, and maternal chronic diseases. The crude RRs and 95% CIs can be seen in eTable 5 in the Supplement.

^c^ The results of model 2, using only twin pregnancy data, with adjusting for geographic region, maternal age, year, ethnicity, and maternal chronic diseases. The crude RRs and 95% CIs can be seen in eTable 5 in the Supplement.

^d^ The results of model 3, using both singleton pregnancy and twin pregnancy data, with adjusting for geographic region, maternal age, year, ethnicity, and maternal chronic diseases. The crude RRs and 95% CIs can be seen in eTable 5 in the Supplement.

^e^ The value of the aRR is significantly higher than 1.00.

^f^ The value of the aRR is significantly lower than 1.00.

^g^ Among a total of 16 879 728 pregnant women aged 20 to 49 years analyzed in this study, there were 1 879 110 women (11.1%) with missing values in birth weight who were not included when performing the description and comparison of low birth weight and very low birth weight.

We also observed 6 categories of neonatal outcomes in this study (Table 2 and eTable 5 in the Supplement). The IVF group had a higher risk than the non-IVF group in fetal growth restriction (aRR, 1.09; 95% CI, 1.06-1.12), low birth weight (aRR, 1.10; 95% CI, 1.09-1.11), very low birth weight (aRR, 1.14; 95% CI, 1.11-1.17), and malformation (aRR, 1.09; 95% CI, 1.06-1.13) but a lower risk of macrosomia (aRR, 0.98; 95% CI, 0.96-1.00) and stillbirth (aRR, 0.68; 95% CI, 0.65-0.71). The models showed a significant interaction between IVF and twin pregnancy for all neonatal outcomes (eTable 6 in the Supplement). Further analyses of twin pregnancies showed that the IVF-T group had higher risk than the nIVF-T group for low birth weight (aRR, 1.03; 95% CI, 1.01-1.04) and very low birth weight (aRR, 1.07; 95% CI, 1.03-1.11) but a lower risk for fetal growth restriction (aRR, 0.96; 95% CI, 0.93-1.00), macrosomia (aRR, 0.35; 95% CI, 0.29-0.43), malformation (aRR, 0.74; 95% CI, 0.70-0.77), and stillbirth (aRR, 0.75; 95% CI, 0.72-0.79). The rate of low birth weight was quite high among twin pregnancies: 43.8% in the IVF-T group and 43.3% in the nIVF-T group. Sensitivity analyses showed that the associations of IVF with maternal and neonatal outcomes were similar in multivariable adjusted analyses stratified by calendar years (before or after 2016) (eTables 7 and 8 in the Supplement).

### Absolute Obstetric Risk by Maternal Age, IVF, and Twin Pregnancy

**Figure 2.**
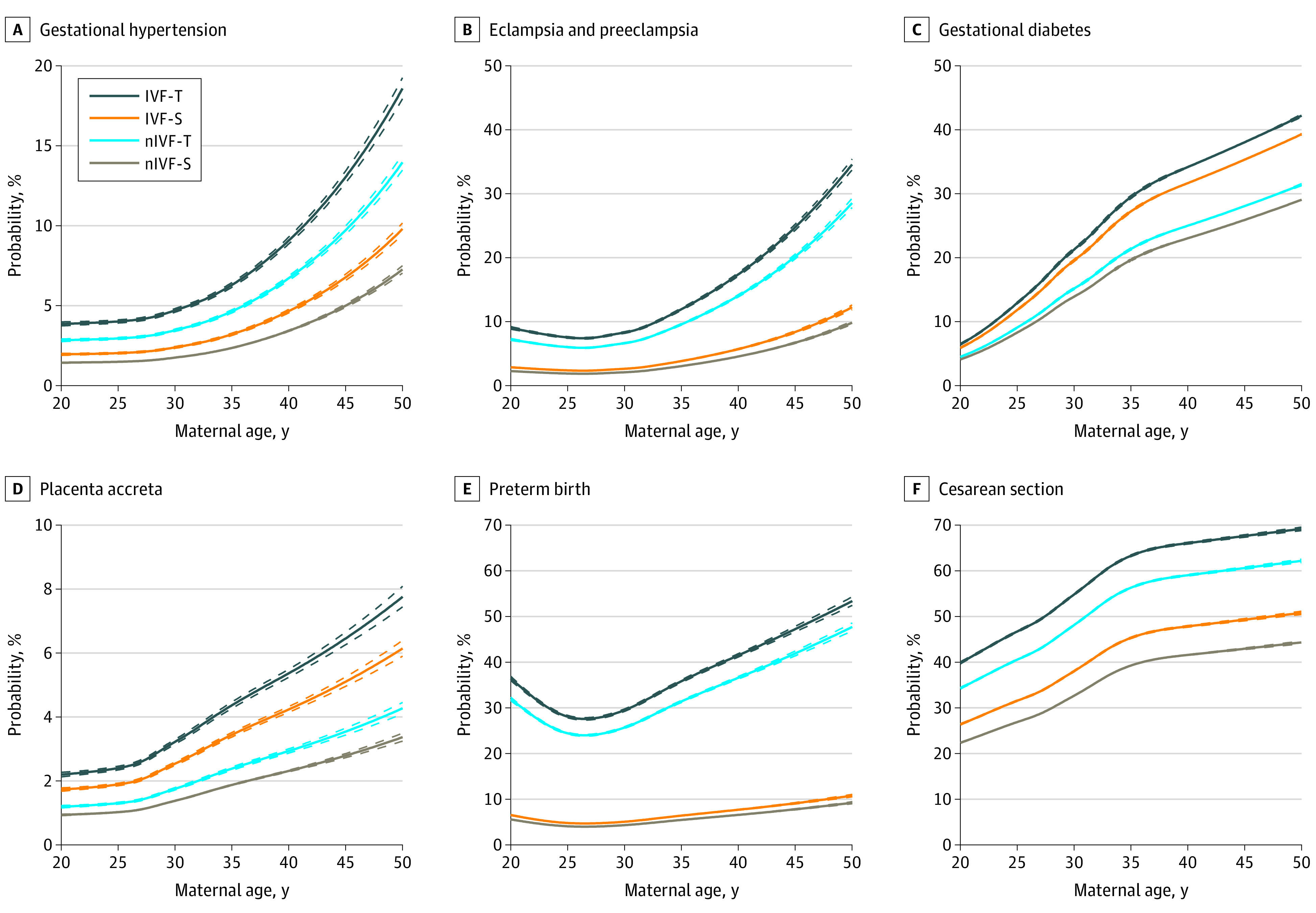
Estimated Absolute Risks of Obstetric Outcomes at Each Maternal Age in Each Subgroup Data are stratified by in vitro fertilization with singleton pregnancy (IVF-S), IVF with twin pregnancy (IVF-T), non-IVF with singleton pregnancy (nIVF-S), and non-IVF with twin pregnancy (nIVF-T). Data are from China, 2013 to 2018. Estimated absolute risks were calculated by using model 3, with singleton pregnancy and twin pregnancy data for risk by maternal age, with adjustment for geographic region, maternal age, year, ethnicity, and maternal chronic diseases. More images can be seen in the eFigure in the Supplement. Detailed data can be seen in eTable 9 in the Supplement. Dashed lines indicate 95% CIs.

Figure 2, accompanied by the eFigure and eTable 9 in the Supplement, presents the estimated absolute risks (probabilities and 95% CIs) of each outcome in each subgroup at each maternal age ranging from 20 to 49 years. These curves show that the obstetric risk in each subgroup was almost always elevated with increasing maternal age. Although the forms of each curve differed, they can be summarized in 2 dominant patterns. Pattern A indicated the absolute risk ranging from IVF-T to nIVF-T to IVF-S to nIVF-S, which presented with gestational hypertension, eclampsia and preeclampsia, placental abruption, preterm birth, dystocia, cesarean delivery, postpartum hemorrhage, fetal growth restriction, low birth weight, very low birth weight, and malformation. Pattern B indicated the absolute risk as ranging from IVF-T to IVF-S to nIVF-T to nIVF-S, which presented with gestational diabetes and placenta accreta. The absolute risk of placenta previa partly complied with pattern B, but there was no difference between singleton and twin pregnancies in the IVF group or non-IVF group at certain intervals of maternal age. In addition, there were 2 sporadic patterns: singleton pregnancy had a higher risk of macrosomia than twin pregnancy, but there was no difference between the IVF group and the non-IVF group; and the risk of stillbirth ranged from nIVF-T to IVF-T to nIVF-S to IVF-S.

## Discussion

Based on national hospital data, we estimated the absolute risk of obstetric outcomes stratified by IVF or non-IVF conception and twin or singleton pregnancy at each maternal age using nonlinear models and found visual evidence suggesting that IVF-conceived twin pregnancies had a higher risk of maternal and neonatal complications than IVF-conceived singleton pregnancies or non–IVF-conceived twin pregnancies. Our findings provide valuable evidence for clinical decision-making and public health policies.

Among IVF-conceived twin pregnancies, the most common obstetric risks were cesarean delivery, low birth weight, preterm birth, gestational diabetes, gestational hypertension, preeclampsia and eclampsia, dystocia, and postpartum hemorrhage, which is consistent with findings of previous studies conducted in varied countries and populations.^6,13,19^ Most curves of obstetric risks showed an elevated trend with increasing maternal age, especially among women older than 35 years, which is also in line with findings of previous literature.^17,18,27,28^ In this study, we further summarized the forms of each obstetric risk curve into 2 dominant patterns. Pattern A presented that at each maternal age, the risk effects of twin pregnancy appeared more prominent than those of IVF, whereas pattern B presented the opposite appearance. The former was more common than the latter, but both revealed that IVF-conceived twin pregnancies had the highest risks in most obstetric outcomes observed in this study. These findings indicate that twin pregnancy, IVF, and advanced maternal age (often defined as ≥35 years^29,30^) are independent indicators for many adverse obstetric outcomes, and their coexistence will lead to the aggravation of obstetric risk.

In addition, 2 sporadic patterns of obstetric risks were present in macrosomia and stillbirth. The absolute risk of macrosomia appeared higher in singleton pregnancies than in twin pregnancies but was not different between the non-IVF group and the IVF group, which is in accordance with previous research.^31,32^ The absolute risk of stillbirth appeared higher in twin pregnancies than in singleton pregnancies, which is also in line with previous literature^9,11^; however, it appeared lower in the IVF group than in the non-IVF group, which differs from previous literature.^31,33^ This may be due to a high compliance with antenatal examinations among pregnant women with IVF conception, especially for noninvasive prenatal testing or amniocentesis.^34,35^ Because artificial abortion is legal in China, pregnancies with abnormalities may be terminated early, which can reduce the incidence of stillbirth reported after gestational age of 20 weeks.

One serious problem revealed in this study was the high rate of twin pregnancy among pregnant women in the IVF group (32.1%). With improvements in IVF technology and increasing recognition of the risk of multiple pregnancies, elective single-embryo transfer (eSET) is recognized as an effective strategy to reduce multiple pregnancies after IVF.^36,37^ For instance, the UK introduced a policy in 2009 to encourage fertility centers to adopt eSET into routine use, which resulted in a sustained trend in the reduction of the multiple pregnancy rate from 26.6% in 2008 to 16.3% in 2013^38^; it then dropped to 6% in 2019.^39^ The US published eSET guidance for patient selection in 2012, recommending that eSET should be offered to patients younger than 35 years,^38^ which led to a persistent reduction in the twin pregnancy rate in the group younger than 35 years from 30.8% in 2011 to 9.9% in 2018.^40^ However, there is no legal regulation or clinical guideline to promote the use of eSET in China; thus, many assisted reproductive centers are not actively promoting eSET, which results in a higher rate of twin pregnancy along with the adverse pregnancy outcomes as mentioned above.

Another noteworthy problem revealed in this study was the high rate of cesarean delivery in each subgroup (IVF-T, 88.8%; nIVF-T, 80.3%; IVF-S, 66.5%; nIVF-S, 43.6%), which was much higher than the global average (21.1%)^41^ or the upper threshold (15%-19%) recommended to reduce maternal and neonatal mortality.^42,43^ Neither IVF nor twin pregnancy themselves are associated with cesarean delivery, if there are no serious complications medically indicating the necessity of cesarean delivery.^44,45,46^ In China, the overuse of cesarean delivery has been driven by complex factors, such as the economic incentives for clinicians, a culture of obstetrician-led delivery, inadequate resources for pain relief in labor, and maternal request with inadequate risk awareness.^47,48^ Therefore, unnecessary cesarean delivery is a major challenge in China, which can cause avoidable harms or unnecessarily increase the need for additional intervention and costs.^47^ There is an urgent need to reduce unnecessary cesarean deliveries to improve maternal and offspring health.

### Limitations

Several limitations must be considered when interpreting the findings of this study. First, there may be selection bias in our study, because pregnant women with serious obstetric complications or higher socioeconomic status are more likely to give birth in tertiary hospitals; thus, it is necessary to further expand the sampling hospitals of the HQMS to different levels with different population groups. Moreover, several crucial factors of obstetric risk were absent in the database of HQMS, such as parity, gestational age, the adoption of risk behaviors (eg, smoking or drinking), paternal characteristics, family history, the reason for cesarean delivery (with any medical indicator or not), the number of embryos transferred and oocyte source in IVF pregnancies, and the chorionicity and amnioticity of the twins; thus, we recommend establishing integrated health information systems for cross-sectoral sharing of data and link them with other medical databases or population databases to retrieve more available and reliable data for promoting evidence-based practices.

## Conclusions

This cohort study estimated the absolute risk of obstetric outcomes at each maternal age among twin pregnancies conceived with IVF in a large Chinese population. The findings suggest that twin pregnancy, IVF, and advanced maternal age are independent indicators of adverse obstetric outcomes, and their coexistence may lead to the aggravation of obstetric risk. We call for developing comprehensive and evidence-based guidelines of health care management for pregnant women with such obstetric characteristics. Furthermore, there is a need to promote eSET to reduce multiple pregnancies after IVF, and unnecessary cesarean delivery should be avoided in all pregnant women.

## Notes

Supplement. **eTable 1.** Variables and the *ICD* Codes **eTable 2.** The Study Population of Subgroups in Each Year (2013-2018) **eTable 3.** The Study Population of Subgroups in Each Province (2013-2018) **eTable 4.** The Study Population of Subgroups at Each Maternal Age in This Study **eTable 5.** Crude RRs and 95% CIs of Obstetric Outcomes Between Different Subgroups **eTable 6.** Interaction Between IVF and Twin Pregnancy for Each Maternal or Neonatal Outcome **eTable 7.** Maternal and Neonatal Outcomes in Subgroups During 2013-2015 and 2016-2018 **eTable 8.** Associations of IVF With Maternal and Neonatal Outcomes in Multivariable Adjusted Analyses Stratified by Calendar Years **eTable 9.** Predicted Absolute Risks of Obstetric Outcomes at Each Maternal Age in Different Subgroups **eFigure.** Predicted Absolute Risks of Obstetric Outcomes at Each Maternal Age in Each Subgroup, China, 2013-2018
