# Supplementary material for: Absolute Risk of Adverse Obstetric Outcomes Among Twin Pregnancies After In Vitro Fertilization by Maternal Age
Source: JAMA Netw Open. 2021 Sep 10;4(9):e2123634. doi: 10.1001/jamanetworkopen.2021.23634 (PMC8433605; doi:10.1001/jamanetworkopen.2021.23634)
Supplement: Supplement. — eTable 1. Variables and the ICD Codes eTable 2. The Study Population of Subgroups in Each Year (2013-2018) eTable 3. The Study Population of Subgroups in Each Province (2013-2018) eTable 4. The Study Population of Subgroups at Each Maternal Age in This Study eTable 5. Crude RRs and 95% CIs of Obstetric Outcomes Between Different Subgroups eTable 6. Interaction Between IVF and Twin Pregnancy for Each Maternal or Neonatal Outcome eTable 7. Maternal and Neonatal Outcomes in Subgroups During 2013-2015 and 2016-2018 eTable 8. Associations of IVF With Maternal and Neonatal Outcomes in Multivariable Adjusted Analyses Stratified by Calendar Years eTable 9. Predicted Absolute Risks of Obstetric Outcomes at Each Maternal Age in Different Subgroups eFigure. Predicted Absolute Risks of Obstetric Outcomes at Each Maternal Age in Each Subgroup, China, 2013-2018 [file jamanetwopen-e2123634-s001.pdf]

Supplementary Online Content

Wang Y, Shi H, Chen L, et al. Absolute risk of adverse obstetric outcomes among twin pregnancies after in vitro fertilization by maternal age. *JAMA Netw Open*. 2021;4(9):e2123634. doi:10.1001/jamanetworkopen.2021.23634

eTable 1. Variables and the *ICD* Codes
eTable 2. The Study Population of Subgroups in Each Year (2013-2018)
eTable 3. The Study Population of Subgroups in Each Province (2013-2018)
eTable 4. The Study Population of Subgroups at Each Maternal Age in This Study
eTable 5. Crude RRs and 95% CIs of Obstetric Outcomes Between Different Subgroups
eTable 6. Interaction Between IVF and Twin Pregnancy for Each Maternal or Neonatal Outcome
eTable 7. Maternal and Neonatal Outcomes in Subgroups During 2013-2015 and 2016-2018
eTable 8. Associations of IVF With Maternal and Neonatal Outcomes in Multivariable Adjusted Analyses Stratified by Calendar Years
eTable 9. Predicted Absolute Risks of Obstetric Outcomes at Each Maternal Age in Different Subgroups
eFigure. Predicted Absolute Risks of Obstetric Outcomes at Each Maternal Age in Each Subgroup, China, 2013-2018

This supplementary material has been provided by the authors to give readers additional information about their work.

©2021 Wang Y et al. *JAMA Network Open*.

eTable 1. Variables and the *ICD* Codes

Variables	ICD codes
**Characteristics of pregnancies**	
In-vitro fertilization (IVF)	Z31.200, Z31.201, Z37.002, Z37.204, Z37.303, Z37.502, 69.9200x004, 69.9200x006, 69.9202
Twin pregnancy	O30.000, O31.201, O35.811, O43.001, O66.100, O66.101, O69.209, P01.501, P02.301, P50.300, P50.500, Z37.200, Z37.201, Z37.202, Z37.203, Z37.204, Z37.300, Z37.301, Z37.302, Z37.303, Z37.400, Z38.300, Z38.400, Z38.500
Singleton pregnancy	Cases except for multiple pregnancies: O30.000, O30.100, O30.200, O30.800, O30.801, O30.900, O31.000, O31.100, O31.200, O31.201, O31.800, O32.500, O32.501, O35.811, O43.001, O63.200, O63.201, O66.100, O66.101, O69.209, O84.000, O84.100, O84.200, O84.800, O84.900, P01.500, P01.501, P01.502, P02.301, P50.300, P50.500, Z37.200, Z37.201, Z37.202, Z37.203, Z37.204, Z37.300, Z37.301, Z37.302, Z37.303, Z37.400, Z37.500, Z37.501, Z37.502, Z37.600, Z37.601, Z37.700, Z38.300, Z38.400, Z38.500, Z38.600, Z38.700, Z38.800
**Maternal chronic diseases (defined as chronic diseases that the pregnant woman had before pregnancy)**	
Chronic hypertension	O10.000, O10.001, O10.400, O10.401, O10.900
Diabetes	O24.000, O24.100, O24.200, O24.300, O24.301
Thyroid diseases	O99.217, O99.216, O99.215, O99.218, O99.219
Anemia	O99.000, O99.003, O99.004, O99.005, O99.006, O99.007
Circulatory diseases	O10.100, O10.101, O10.300, O10.301, O99.400, O99.419, O99.420, O99.428, O99.429, O99.401, O99.432, O99.430, O99.431, O99.433, O99.409, O90.300, O99.415, O99.408, O99.421, O99.413, O99.416, O99.417, O99.414, O99.402, O75.403, O99.403, O99.418, O99.422, O99.412, O99.410, O99.423, O99.424, O99.406, O99.407, O99.411, O99.404, O99.405
Other diseases (coagulation disorders, kidney diseases, diseases of connective tissues, diseases of respiratory system, and diseases of digestive system)	O99.002, O99.101, O99.102, O99.103, O99.104, O99.105, O99.106, O99.107, O99.108, O99.100, O26.801, O26.802, O90.400, O26.803, O26.804, O26.805, O23.000, O23.001, O23.100, O23.101, O23.200, O23.300, O23.400, O23.900, O23.901, O99.806, O86.200, O86.201, O86.300, O99.811, O99.812, O99.109, O99.805, O99.813, O99.501, O99.502, O99.503, O99.504, O99.505, O99.506, O99.510, O99.511, O99.512, O99.507, O99.509, O99.508, O99.500, O99.600, O99.601, O99.602, O99.603, O99.604, O99.605, O99.606, O99.607, O99.608, O99.609, O99.610, O99.611, O99.612, O99.613, O99.614, O99.615, O99.616, O99.617, O99.618, O99.619, O99.620, O99.621, O99.622, O99.623, O99.624, O26.600, O26.601, O26.602, O26.603, O26.604, O26.605
**Obstetric outcomes**	
Maternal outcomes (defined as maternal complications that developed during pregnancy)	
Gestational hypertension	O16.x00, O12.000, O12.100, O12.200, O13.x00, O13.x01

©2021 Wang Y et al. *JAMA Network Open*.

Variables	ICD codes
Preeclampsia & eclampsia	O11.x00, O11.x01, O14.101, O13.x02, O14.000, O14.100, O14.102, O14.900, O15.000, O15.001, O15.100, O15.101, O15.200, O15.201, O15.900
Gestational diabetes	O24.400, O24.900
Placenta previa	O44.000, O44.001, O44.002, O44.003, O44.100, O44.101, O44.102, O44.103
Placental abruption	O45.000, O45.001, O45.800, O45.801, O45.900
Placenta accrete	O72.001
Preterm	O60.000, O60.001, O60.100, O60.300, P07.300, P59.000, P61.200
Dystocia	O64.000, O64.001, O64.002, O64.003, O64.100, O64.101, O64.200, O64.300, O64.301, O64.400, O64.401, O64.500, O64.501, O64.800, O64.801, O64.802, O64.803, O64.804, O64.805, O64.900, O32.000, O32.100, O32.101, O32.102, O32.200, O32.201, O32.202, O32.300, O32.301, O32.302, O32.303, O32.400, O32.500, O32.501, O32.600, O32.601, O32.800, O32.801, O32.802, O32.803, O32.900, O65.000, O65.001, O65.100, O65.101, O65.200, O65.201, O65.202, O65.300, O65.301, O33.000, O33.001, O33.002, O33.100, O33.101, O33.102, O33.200, O33.201, O33.300, O33.301, O65.800, O65.801, O65.900, O65.400, O65.401, O33.400, O33.500, O33.600, O33.700, O33.800, O33.900, O66.800, O66.900, O66.901
Caesarean section	O66.401, O82.000, O82.100, O82.200, O82.800, O82.900, O84.200, P03.401, O86.002, O90.000, P03.400, 74.0 001, 74.1 001, 74.1 002, 74.1 003, 74.2 002, 74.4 003
Postpartum hemorrhage	O72.001, O72.002, O72.201, O72.101, O72.202, O72.000, O72.100, O72.200
Neonatal outcomes (defined as neonatal complications that developed before or after birth until the discharge)	
Fetal growth restriction (FGR)	O36.500, O36.504, O36.503, P05.000, P05.100, P05.101, P05.102, P05.200, P05.201, P05.900
Low birth weight	Calculated according to birth weight records (birth weight <2500g)
Very low birth weight	Calculated according to birth weight records (birth weight <1500g)
Macrosomia	O33.500, O33.501, O36.600, O36.601, O66.200, O66.201, O66.202, P08.000, P08.100
Malformation	O35.000, O35.001, O35.002, O35.003, O35.004, O35.005, O35.006, O35.007, O35.008, O35.009, O35.100, O35.101, O35.102, O35.200, O35.201, O35.202, O35.203, O35.204, O35.205, O35.206, O28.001, O35.800, O35.801, O35.802, O35.803, O35.804, O35.805, O35.806, O35.807, O35.808, O35.809, O35.810, O35.811, O35.812, O35.813, O35.814, O35.815, O35.816, O35.817, O35.818, O35.819, O35.820, O35.821, O35.822, O35.823, O35.900, O36.200, O36.201
Stillbirth	O36.400, O36.401, O31.200, O31.201, P95.x00, Z37.100, Z37.300, Z37.302, Z37.303, Z37.601, Z37.400, Z37.700

©2021 Wang Y et al. *JAMA Network Open*.

eTable 2. The Study Population of Subgroups in Each Year (2013-2018)

Subgroup	2013	2014	2015	2016	2017	2018
nIVF_S	746520 (97.0%)	1428376 (97.1%)	1314390 (96.6%)	4328514 (97.2%)	4403157 (97.0%)	4131836 (96.4%)
nIVF_T	15135 (2.0%)	28014 (1.9%)	25494 (1.9%)	64329 (1.4%)	64565 (1.4%)	59660 (1.4%)
IVF_S	5477 (0.7%)	9791 (0.7%)	13383 (1.0%)	41992 (0.9%)	47874 (1.1%)	64542 (1.5%)
IVF_T	2855 (0.4%)	5438 (0.4%)	7546 (0.6%)	19341 (0.4%)	22509 (0.5%)	28990 (0.7%)
**Total**	**769987 (100.0%)**	**1471619 (100.0%)**	**1360813 (100.0%)**	**4454176 (100.0%)**	**4538105 (100.0%)**	**4285028 (100.0%)**

©2021 Wang Y et al. *JAMA Network Open*.

eTable 3. The Study Population of Subgroups in Each Province (2013-2018)

	2013					2014					2015			
	nIVF_S	nIVF_T	IVF_S	IVF_T		nIVF_S	nIVF_T	IVF_S	IVF_T		nIVF_S	nIVF_T	IVF_S	IVF_T
**Eastern region**	**434657**	**8601**	**3453**	**1852**		**762927**	**14621**	**5908**	**3443**		**675634**	**12676**	**8131**	**4438**
Beijing	50785	762	961	433		70020	1213	832	450		50203	1115	813	348
Tianjin	3633	71	8	3		10224	140	19	8		8819	88	26	15
Hebei	15807	449	40	34		27031	723	100	58		22885	514	197	110
Liaoning	13235	402	96	52		26706	709	221	176		19054	483	283	199
Shanghai	64165	1221	730	390		83917	1288	967	441		72766	1219	1396	707
Jiangsu	82082	1472	354	145		149500	2650	902	397		125656	2226	1229	526
Zhejiang	32816	689	253	209		64923	1220	554	448		51926	1019	728	467
Fujian	21716	258	93	39		63354	919	420	273		57768	839	417	255
Shandong	59133	1816	172	98		104870	3158	346	190		76449	2142	291	110
Guangdong	84845	1286	690	410		152495	2376	1418	890		179870	2850	2585	1604
Hainan	6440	175	56	39		9887	225	129	112		10238	181	166	97
**Central region**	**145401**	**3226**	**596**	**339**		**342354**	**7109**	**1528**	**975**		**325294**	**6562**	**2305**	**1501**
Shanxi	9066	243	16	8		40417	1156	56	32		29249	761	150	99
Jilin	4660	151	13	23		8054	236	64	62		7569	215	114	72
Heilongjiang	5268	83	4	1		16518	288	112	63		10424	181	105	64
Anhui	28593	643	81	43		53030	1026	164	60		46162	794	192	106
Jiangxi	15103	205	72	32		31666	407	161	77		36526	466	229	136
Henan	20439	626	34	19		70634	1857	108	82		59578	1713	161	122
Hubei	53493	1131	254	162		103625	1766	462	338		111493	2001	855	559
Hunan	8779	144	122	51		18410	373	401	261		24293	431	499	343
**Western region**	**166462**	**3308**	**1428**	**664**		**323095**	**6284**	**2355**	**1020**		**313462**	**6256**	**2947**	**1607**
Sichuan	33072	677	290	208		80390	1526	372	192		82390	1448	706	490
Chongqing	6945	178	54	45		11443	265	59	66		18865	481	203	186
Guizhou	15745	334	39	28		28653	560	140	99		26235	570	121	87

©2021 Wang Y et al. *JAMA Network Open*.

Yunnan	30305	662	445	197		62499	1298	661	323		59248	1251	954	460
Tibet	41	0	0	0		0	0	0	0		0	0	0	0
Shaanxi	10678	183	245	8		33009	627	461	19		30671	676	54	24
Gansu	4549	26	49	4		6345	53	25	14		6153	76	48	19
Qinghai	6932	125	8	0		12075	237	28	11		10181	197	57	19
Ningxia	2249	50	2	2		2313	64	2	0		3803	112	23	8
Xinjiang	8301	244	5	4		12786	381	19	8		12719	385	58	21
Guangxi	22017	382	206	119		34987	640	398	215		38641	663	506	236
Inner Mongolia	25628	447	85	49		38595	633	190	73		24556	397	217	57

nIVF_S: singleton pregnancy conceived with non-IVF; nIVF_T: twin pregnancy conceived with non-IVF; IVF_S: singleton pregnancy conceived with IVF; IVF_T: twin pregnancy conceived with IVF.

eTable 3: ***(Continued)***

	**2016**					**2017**					**2018**			
	**nIVF_S**	**nIVF_T**	**IVF_S**	**IVF_T**		**nIVF_S**	**nIVF_T**	**IVF_S**	**IVF_T**		**nIVF_S**	**nIVF_T**	**IVF_S**	**IVF_T**
**Eastern region**	**2205831**	**33413**	**20170**	**10715**		**2237463**	**32567**	**28019**	**12453**		**2044876**	**29591**	**35572**	**15024**
Beijing	106434	1403	1745	675		96368	1315	2296	824		90385	1198	3028	1044
Tianjin	57738	783	527	284		58541	742	840	395		51157	652	884	338
Hebei	137544	2330	797	399		128221	2054	1099	455		112699	1887	1363	542
Liaoning	110383	1077	572	282		100110	1091	703	292		93780	1130	1052	375
Shanghai	102706	1180	2121	946		89033	1023	2752	1138		80015	1271	3454	1336
Jiangsu	326334	4830	2946	1372		313916	4571	4010	1631		298416	4359	4932	1872
Zhejiang	300944	3516	2568	1562		314443	3685	3578	1798		281055	3342	4483	2218
Fujian	167282	2803	1510	683		179945	2798	1617	712		155438	2259	2002	759
Shandong	356222	6922	1729	789		374423	6841	3522	1050		344160	5673	4268	1606
Guangdong	496682	8046	5239	3528		533089	7919	7027	3920		489709	7303	9310	4659
Hainan	43562	523	416	195		49374	528	575	238		48062	517	796	275
**Central region**	**1018896**	**14441**	**13954**	**4920**		**1019203**	**14518**	**10505**	**5673**		**957762**	**12699**	**15077**	**7276**

Shanxi	102820	1747	398	291		93870	1547	691	316		96416	1627	979	499
Jilin	64272	617	254	108		57319	716	353	92		51344	590	440	245
Heilongjiang	57587	521	533	167		50717	505	661	182		51229	524	710	162
Anhui	134130	2186	863	620		146741	2348	1224	751		140603	1986	1676	801
Jiangxi	123584	1919	1391	1112		132506	2062	1776	1278		118271	1457	2197	1479
Henan	206099	3814	562	453		194240	3618	959	614		182238	3007	1544	940
Hubei	167154	2042	1434	930		168866	2197	2116	1124		159991	2044	4140	1579
Hunan	163250	1595	8519	1239		174944	1525	2725	1316		157670	1464	3391	1571
**Western region**	**1103787**	**16475**	**7868**	**3706**		**1146491**	**17480**	**9350**	**4383**		**1129198**	**17370**	**13893**	**6690**
Sichuan	286397	4417	2481	1105		282382	4939	2301	1250		265251	4957	3161	1851
Chongqing	61718	871	627	448		63619	1061	705	507		61134	976	880	612
Guizhou	108079	1449	456	262		132069	1824	586	325		135096	2065	852	548
Yunnan	100974	1274	1135	407		113024	1422	1419	479		109029	1701	2150	1055
Tibet	5572	51	2	0		5729	59	2	1		9131	127	13	2
Shaanxi	127596	2238	300	125		131493	2265	452	208		136746	2206	695	323
Gansu	50508	861	169	125		49908	923	201	140		62713	823	1279	358
Qinghai	19290	267	80	45		19609	264	135	65		20954	246	203	97
Ningxia	24324	440	85	32		22637	391	100	23		26427	364	218	95
Xinjiang	81137	1461	345	148		78136	1157	504	231		75461	1003	635	281
Guangxi	163843	2488	1761	858		181413	2572	2460	1012		160514	2204	3132	1230
Inner Mongolia	74349	658	427	151		66472	603	485	142		66742	698	675	238

nIVF_S: singleton pregnancy conceived with non-IVF; nIVF_T: twin pregnancy conceived with non-IVF; IVF_S: singleton pregnancy conceived with IVF; IVF_T: twin pregnancy conceived with IVF.

©2021 Wang Y et al. *JAMA Network Open*.

eTable 4. The Study Population of Subgroups at Each Maternal Age in This Study

Maternal age (year)	nIVF_S	nIVF_T	IVF_S	IVF_T
20	203281	3125	214	31
21	306523	4582	424	127
22	437194	6401	738	318
23	621670	9298	1288	648
24	846287	12439	2195	1293
25	1071775	15416	3647	2176
26	1370437	19294	5909	3616
27	1583484	22509	8356	5217
28	1639927	24021	11296	6944
29	1476327	22578	13825	8092
30	1225605	20141	14950	8474
31	1059901	18234	16277	8668
32	885803	15693	15647	8213
33	735248	13455	15199	7026
34	658636	11600	14654	6432
35	545460	9699	13246	5391
36	445917	8019	11504	4342
37	340731	6097	8952	3100
38	271890	4642	7030	2196
39	207632	3498	5598	1517
40	152073	2354	4297	960
41	104563	1588	2956	640
42	68885	1021	1920	369
43	42431	594	1147	226
44	24054	352	670	145
45	12793	206	427	126
46	6612	112	243	125
47	3611	111	210	97
48	2335	71	139	98
49	1708	47	101	72

nIVF_S: singleton pregnancy conceived with non-IVF; nIVF_T: twin pregnancy conceived with non-IVF; IVF_S: singleton pregnancy conceived with IVF; IVF_T: twin pregnancy conceived with IVF.

©2021 Wang Y et al. *JAMA Network Open*.

eTable 5. Crude RRs and 95% CIs of Obstetric Outcomes Between Different Subgroups

	Crude RR (95% CI) #		
	**IVF_S vs nIVF_S**	**IVF_T vs nIVF_T**	**IVF vs nIVF**
**Maternal outcomes**			
Gestational hypertension	**1.97 (1.92 to 2.02)**	**1.25 (1.21 to 1.30)**	**2.19 (2.15 to 2.24)**
Eclampsia & preeclampsia	**1.97 (1.94 to 2.01)**	**1.15 (1.13 to 1.18)**	**2.67 (2.63 to 2.70)**
Gestational diabetes	**2.05 (2.04 to 2.07)**	**1.71 (1.68 to 1.74)**	**2.03 (2.01 to 2.05)**
Placenta previa	**2.48 (2.42 to 2.53)**	**1.86 (1.79 to 1.94)**	**2.34 (2.30 to 2.38)**
Placental abruption	**1.32 (1.26 to 1.38)**	**1.07 (1.00 to 1.15)**	**1.44 (1.39 to 1.49)**
Placenta accreta	**2.40 (2.35 to 2.45)**	**1.62 (1.56 to 1.69)**	**2.39 (2.35 to 2.43)**
Preterm birth	**1.70 (1.67 to 1.72)**	**1.10 (1.09 to 1.11)**	**3.44 (3.41 to 3.47)**
Dystocia	**1.32 (1.30 to 1.34)**	**1.30 (1.28 to 1.33)**	**1.52 (1.50 to 1.53)**
Cesarean section	**1.53 (1.52 to 1.53)**	**1.11 (1.10 to 1.11)**	**1.67 (1.66 to 1.67)**
Postpartum hemorrhage	**1.98 (1.95 to 2.01)**	**1.48 (1.45 to 1.51)**	**2.27 (2.24 to 2.30)**
**Neonatal outcomes**			
FGR	**1.49 (1.43 to 1.55)**	1.00 (0.97 to 1.03)	**2.86 (2.79 to 2.93)**
Low birth weight *	**1.44 (1.41 to 1.46)**	**1.01 (1.00 to 1.02)**	**3.35 (3.32 to 3.38)**
Very low birth weight *	**1.60 (1.54 to 1.67)**	1.01 (0.97 to 1.05)	**2.62 (2.56 to 2.69)**
Macrosomia	**1.11 (1.09 to 1.13)**	***0.35 (0.29 to 0.43)***	***0.77 (0.76 to 0.79)***
Malformation	**1.74 (1.68 to 1.80)**	***0.79 (0.75 to 0.82)***	**2.00 (1.95 to 2.05)**
Stillbirth	***0.51 (0.46 to 0.56)***	***0.73 (0.70 to 0.77)***	**2.31 (2.22 to 2.40)**

FGR: fetal growth restriction.

* Among a total of 16,879,728 pregnant women aged 20-49 years analyzed in this study, there were 1,879,110 women (11.1%) with missing values in *"birth weight"*, which were not included when performing the description and comparison of "low birth weight" and "very low birth weight".

Bold number refers to the value of RR significantly higher than 1, and ***Bold and italic number*** refers to the value of RR significantly lower than 1. The adjusted RRs and 95% CIs can be seen in Table 2.

©2021 Wang Y et al. *JAMA Network Open*.

eTable 6. Interaction Between IVF and Twin Pregnancy for Each Maternal or Neonatal Outcome

	aRR (95% CI) [#]			***P*** Value for interaction [&]
	**IVF_S vs nIVF_S**	**IVF_T vs nIVF_T**	**IVF vs nIVF**	
**Maternal outcomes**				
Gestational hypertension	**1.55 (1.51 to 1.59)**	**1.14 (1.10 to 1.19)**	**1.56 (1.52 to 1.60)**	**<0.001**
Eclampsia & preeclampsia	**1.52 (1.49 to 1.55)**	**1.12 (1.09 to 1.14)**	**1.54 (1.51 to 1.57)**	**<0.001**
Gestational diabetes	**1.48 (1.47 to 1.50)**	**1.39 (1.36 to 1.42)**	**1.48 (1.47 to 1.50)**	**<0.001**
Placenta previa	**1.87 (1.83 to 1.91)**	**1.62 (1.55 to 1.69)**	**1.87 (1.83 to 1.91)**	**<0.001**
Placental abruption	**1.16 (1.11 to 1.21)**	**1.03 (0.96 to 1.10)**	**1.16 (1.11 to 1.22)**	**0.001**
Placenta accreta	**2.00 (1.96 to 2.04)**	**1.46 (1.40 to 1.52)**	**2.00 (1.96 to 2.05)**	**<0.001**
Preterm birth	**1.48 (1.46 to 1.51)**	**1.08 (1.07 to 1.10)**	**1.51 (1.48 to 1.53)**	**<0.001**
Dystocia	**1.34 (1.32 to 1.36)**	**1.22 (1.20 to 1.25)**	**1.33 (1.31 to 1.35)**	0.573
Cesarean section	**1.32 (1.31 to 1.33)**	**1.08 (1.07 to 1.09)**	**1.32 (1.32 to 1.33)**	**<0.001**
Postpartum hemorrhage	**1.77 (1.74 to 1.80)**	**1.43 (1.40 to 1.47)**	**1.77 (1.74 to 1.80)**	**<0.001**
**Neonatal outcomes**				
FGR	**1.36 (1.30 to 1.42)**	0.96 (0.93 to 1.00)	**1.38 (1.32 to 1.44)**	**<0.001**
Low birth weight [*]	**1.35 (1.32 to 1.37)**	**1.03 (1.01 to 1.04)**	**1.37 (1.34 to 1.39)**	**<0.001**
Very low birth weight [*]	**1.42 (1.36 to 1.48)**	**1.07 (1.03 to 1.11)**	**1.44 (1.38 to 1.51)**	**<0.001**
Macrosomia	0.99 (0.97 to 1.01)	***0.35 (0.29 to 0.43)***	0.99 (0.98 to 1.01)	**<0.001**
Malformation	**1.53 (1.48 to 1.58)**	***0.74 (0.70 to 0.77)***	**1.53 (1.48 to 1.59)**	**<0.001**
Stillbirth	***0.46 (0.42 to 0.51)***	***0.75 (0.72 to 0.79)***	***0.47 (0.42 to 0.52)***	**<0.001**

FGR: fetal growth restriction.

[*] Among a total of 16,879,728 pregnant women aged 20-49 years analyzed in this study, there were 1,879,110 women (11.1%) with missing values in *"birth weight"*, who were not included when performing the description and comparison of "low birth weight" and "very low birth weight".

[#] aRR (95% CI): **Bold number** refers to the value of RR significantly higher than 1, and ***Bold and italic number*** refers to the value of RR significantly lower than 1.

[&] *P* Value for interaction: the significance of the interaction effect between IVF and twin pregnancy tested by including an interaction term in Model 3.

©2021 Wang Y et al. *JAMA Network Open*.

eTable 7. Maternal and Neonatal Outcomes in Subgroups During 2013-2015 and 2016-2018

	2013-2015						2016-2018					
	Singleton		Twin		Sub-total		Singleton		Twin		Sub-total	
	nIVF	IVF	nIVF	IVF	nIVF	IVF	nIVF	IVF	nIVF	IVF	nIVF	IVF
**Maternal outcomes**												
Gestational hypertension	48227 (1.4)	819 (2.9)	2415 (3.5)	662 (4.2)	50642 (1.4)	1481 (3.3)	221947 (1.7)	5143 (3.3)	7036 (3.7)	3335 (4.7)	228983 (1.8)	8478 (3.8)
Eclampsia & preeclampsia	100574 (2.9)	1382 (4.8)	8267 (12.0)	1863 (11.8)	108841 (3.1)	3245 (7.3)	365086 (2.8)	8902 (5.8)	20425 (10.8)	9271 (13.1)	385511 (3.0)	18173 (8.1)
Gestational diabetes	275673 (7.9)	5871 (20.5)	6525 (9.5)	3119 (19.7)	282198 (7.9)	8990 (20.2)	1402245 (10.9)	32700 (21.2)	24230 (12.9)	14636 (20.7)	1426475 (10.9)	47336 (21.0)
Placenta previa	72633 (2.1)	1554 (5.4)	1637 (2.4)	703 (4.4)	74270 (2.1)	2257 (5.1)	247275 (1.9)	7311 (4.7)	3959 (2.1)	2808 (4.0)	251234 (1.9)	10119 (4.5)
Placental abruption	22795 (0.7)	263 (0.9)	769 (1.1)	199 (1.3)	23564 (0.7)	462 (1.0)	102779 (0.8)	1590 (1.0)	2412 (1.3)	952 (1.3)	105191 (0.8)	2542 (1.1)
Placenta accreta	57402 (1.6)	949 (3.3)	1596 (2.3)	501 (3.2)	58998 (1.7)	1450 (3.3)	260919 (2.0)	7605 (4.9)	5899 (3.1)	3600 (5.1)	266818 (2.0)	11205 (5.0)
Preterm birth	167102 (4.8)	2247 (7.8)	22824 (33.3)	5904 (37.3)	189926 (5.3)	8151 (18.3)	625895 (4.9)	12819 (8.3)	69593 (36.9)	28457 (40.2)	695488 (5.3)	41276 (18.3)
Dystocia	350770 (10.1)	3656 (12.8)	7261 (10.6)	2531 (16.0)	358031 (10.1)	6187 (13.9)	1045032 (8.1)	16974 (11.0)	25985 (13.8)	12053 (17.0)	1071017 (8.2)	29027 (12.9)
Cesarean section	1561428 (44.7)	19430 (67.8)	53351 (77.7)	13169 (83.1)	1614779 (45.4)	32599 (73.3)	5566562 (43.3)	102312 (66.3)	153273 (81.3)	63796 (90.1)	5719835 (43.8)	166108 (73.7)
Postpartum hemorrhage	122771 (3.5)	1909 (6.7)	5949 (8.7)	1982 (12.5)	128720 (3.6)	3891 (8.7)	526845 (4.1)	12494 (8.1)	14748 (7.8)	8333 (11.8)	541593 (4.1)	20827 (9.2)
**Neonatal outcomes**												
FGR	28343 (0.8)	328 (1.1)	2698 (3.9)	703 (4.4)	31041 (0.9)	1031 (2.3)	104174 (0.8)	1877 (1.2)	10868 (5.8)	3874 (5.5)	115042 (0.9)	5751 (2.6)
Low birth weight *	174789 (5.1)	2043 (7.2)	29569 (47.3)	7428 (49.4)	204358 (5.9)	9471 (21.8)	546920 (4.9)	9645 (7.1)	63908 (41.6)	24894 (42.3)	610828 (5.4)	34539 (17.8)
Very low birth weight *	20911 (0.6)	371 (1.3)	3209 (5.1)	832 (5.5)	24120 (0.7)	1203 (2.8)	105691 (1.0)	1914 (1.4)	6919 (4.5)	2660 (4.5)	112610 (1.0)	4574 (2.4)
Macrosomia	183231 (5.3)	1584 (5.5)	141 (0.2)	25 (0.2)	183372 (5.2)	1609 (3.6)	685150 (5.3)	9217 (6.0)	753 (0.4)	81 (0.1)	685903 (5.3)	9298 (4.1)
Malformation	35145 (1.0)	510 (1.8)	1861 (2.7)	403 (2.5)	37006 (1.0)	913 (2.1)	135772 (1.1)	2820 (1.8)	7683 (4.1)	2128 (3.0)	143455 (1.1)	4948 (2.2)
Stillbirth	14516 (0.4)	52 (0.2)	2370 (3.5)	445 (2.8)	16886 (0.5)	497 (1.1)	46757 (0.4)	295 (0.2)	6920 (3.7)	1850 (2.6)	53677 (0.4)	2145 (1.0)

* There were 72049 cases (65057 singletons and 6992 twins) born in 2013-2015 and 1807061 cases (1760116 singletons and 46945 twins) born in 2016-2018 with missing values in *"birth weight"*, who were not included when performing the description and comparison of "low birth weight" and "very low birth weight".

©2021 Wang Y et al. *JAMA Network Open*.

eTable 8. Associations of IVF With Maternal and Neonatal Outcomes in Multivariable Adjusted Analyses Stratified by Calendar Years

	aRR (95% CI) IVF vs. Non-IVF					
	Singleton		Twin		Total	
	2013-2015 (n = 3517937)	2016-2018 (n = 13017915)	2013-2015 (n = 84482)	2016-2018 (n = 259394)	2013-2015 (n = 3602419)	2016-2018 (n = 13277309)
**Maternal outcomes**						
Gestational hypertension	**1.54 (1.43 to 1.65)**	**1.55 (1.50 to 1.59)**	1.08 (0.99 to 1.18)	**1.16 (1.11 to 1.21)**	**1.28 (1.21 to 1.36)**	**1.39 (1.35 to 1.42)**
Eclampsia & preeclampsia	**1.25 (1.18 to 1.32)**	**1.58 (1.54 to 1.61)**	0.97 (0.92 to 1.03)	**1.16 (1.13 to 1.19)**	**1.02 (0.98 to 1.06)**	**1.33 (1.30 to 1.35)**
Gestational diabetes	**1.70 (1.66 to 1.75)**	**1.44 (1.43 to 1.46)**	**1.54 (1.47 to 1.61)**	**1.35 (1.32 to 1.38)**	**1.65 (1.62 to 1.69)**	**1.42 (1.40 to 1.43)**
Placenta previa	**1.80 (1.71 to 1.90)**	**1.88 (1.84 to 1.92)**	**1.51 (1.38 to 1.65)**	**1.64 (1.56 to 1.73)**	**1.73 (1.66 to 1.81)**	**1.83 (1.79 to 1.87)**
Placental abruption	**1.21 (1.07 to 1.36)**	**1.15 (1.10 to 1.21)**	1.12 (0.95 to 1.32)	1.01 (0.94 to 1.09)	**1.15 (1.04 to 1.27)**	**1.10 (1.06 to 1.15)**
Placenta accreta	**1.63 (1.52 to 1.73)**	**2.07 (2.02 to 2.12)**	**1.22 (1.10 to 1.35)**	**1.51 (1.45 to 1.58)**	**1.49 (1.41 to 1.58)**	**1.92 (1.88 to 1.96)**
Preterm	**1.40 (1.34 to 1.46)**	**1.50 (1.47 to 1.53)**	**1.09 (1.06 to 1.12)**	**1.08 (1.06 to 1.09)**	**1.15 (1.12 to 1.17)**	**1.18 (1.16 to 1.19)**
Dystocia	**1.29 (1.25 to 1.33)**	**1.35 (1.33 to 1.37)**	**1.36 (1.30 to 1.43)**	**1.19 (1.17 to 1.22)**	**1.36 (1.32 to 1.39)**	**1.31 (1.29 to 1.33)**
Caesarean section	**1.33 (1.32 to 1.35)**	**1.32 (1.31 to 1.33)**	**1.06 (1.04 to 1.08)**	**1.08 (1.07 to 1.09)**	**1.20 (1.19 to 1.22)**	**1.21 (1.21 to 1.22)**
Postpartum hemorrhage	**1.58 (1.51 to 1.66)**	**1.80 (1.77 to 1.84)**	**1.34 (1.28 to 1.42)**	**1.45 (1.41 to 1.49)**	**1.45 (1.41 to 1.51)**	**1.68 (1.66 to 1.71)**
**Neonatal outcomes**						
FGR	**1.23 (1.10 to 1.37)**	**1.38 (1.32 to 1.45)**	1.08 (0.99 to 1.18)	***0.94 (0.90 to 0.97)***	**1.11 (1.04 to 1.19)**	**1.06 (1.03 to 1.10)**
Low birth weight *	**1.29 (1.24 to 1.35)**	**1.36 (1.34 to 1.39)**	**1.06 (1.03 to 1.09)**	**1.02 (1.00 to 1.03)**	**1.10 (1.07 to 1.12)**	**1.11 (1.10 to 1.12)**
Very low birth weight *	**1.65 (1.49 to 1.83)**	**1.39 (1.33 to 1.45)**	**1.11 (1.03 to 1.20)**	**1.05 (1.01 to 1.11)**	**1.16 (1.09 to 1.24)**	**1.17 (1.13 to 1.21)**
Macrosomia	***0.94 (0.90 to 0.99)***	1.00 (0.98 to 1.02)	***0.63 (0.41 to 0.97)***	***0.31 (0.25 to 0.40)***	***0.94 (0.89 to 0.98)***	**0.99 (0.97 to 1.01)**
Malformation	**1.48 (1.36 to 1.62)**	**1.53 (1.48 to 1.59)**	***0.86 (0.77 to 0.96)***	***0.71 (0.68 to 0.75)***	**1.14 (1.06 to 1.22)**	**1.07 (1.04 to 1.10)**
Stillbirth	***0.38 (0.29 to 0.50)***	***0.48 (0.43 to 0.54)***	***0.89 (0.80 to 0.99)***	***0.72 (0.69 to 0.76)***	***0.73 (0.67 to 0.81)***	***0.65 (0.62 to 0.69)***

FGR: fetal growth restriction.

* There were 72049 cases (65057 singletons and 6992 twins) born in 2013-2015 and 1807061 cases (1760116 singletons and 46945 twins) born in 2016-2018 with missing values in *"birth weight"*, who were not included when performing the description and comparison of "low birth weight" and "very low birth weight".

©2021 Wang Y et al. *JAMA Network Open*.

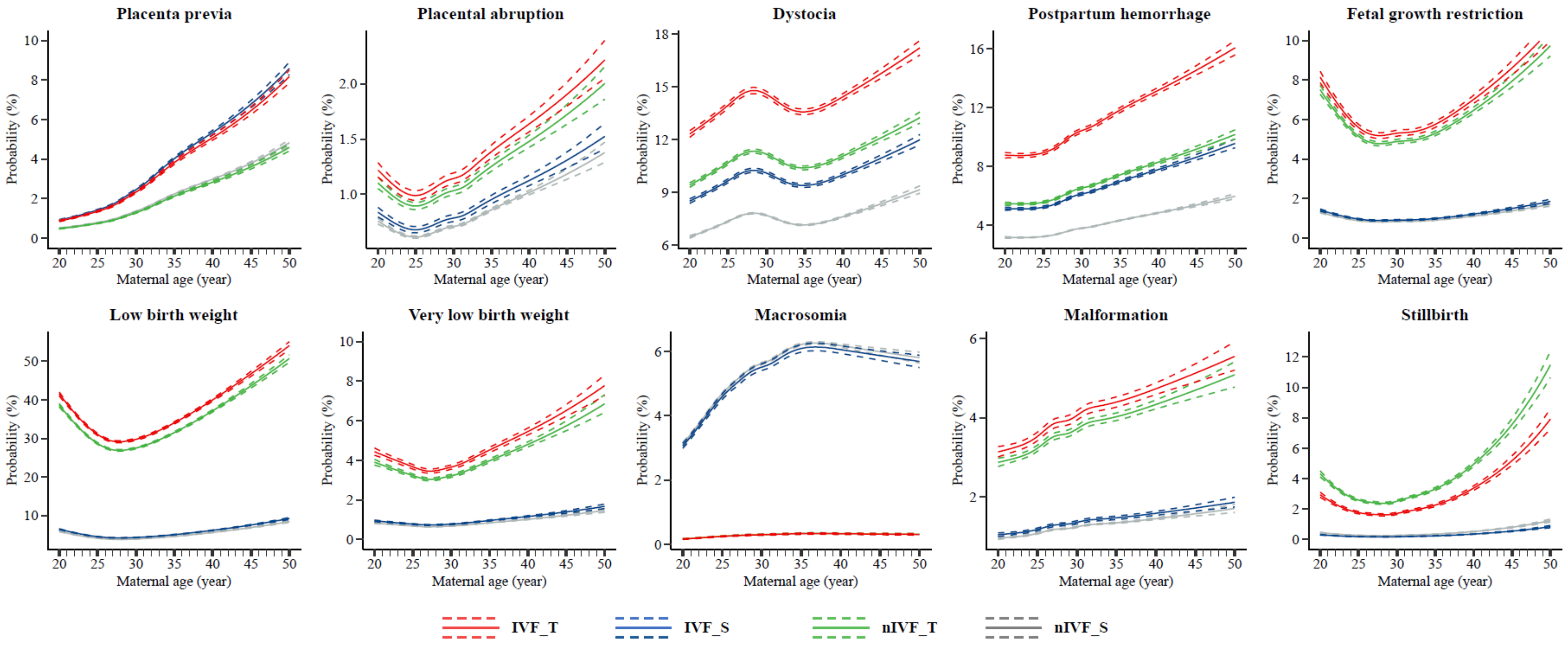

eFigure. Predicted Absolute Risks of Obstetric Outcomes at Each Maternal Age in Each Subgroup, China, 2013-2018

Predicted absolute risks were calculated by using Model 3. nIVF_S: singleton pregnancy conceived with non-IVF; nIVF_T: twin pregnancy conceived with non-IVF; IVF_S: singleton pregnancy conceived with IVF; IVF_T: twin pregnancy conceived with IVF. Other images can be seen in Figure 2. Detail data can be seen in eTable 9 in the Supplement.

©2021 Wang Y et al. *JAMA Network Open*.

eTable 9. Predicted Absolute Risks of Obstetric Outcomes at Each Maternal Age in Different Subgroups

**Gestational hypertension**				
Maternal age (years)	nIVF_S	IVF_S	nIVF_T	IVF_T
20	1.43 (0.01 to 1.47)	1.95 (0.02 to 2.02)	2.84 (0.03 to 2.92)	3.86 (0.04 to 3.98)
21	1.44 (0.01 to 1.47)	1.97 (0.02 to 2.02)	2.85 (0.03 to 2.93)	3.88 (0.04 to 4.00)
22	1.45 (0.01 to 1.47)	1.98 (0.02 to 2.03)	2.87 (0.03 to 2.94)	3.91 (0.04 to 4.01)
23	1.46 (0.01 to 1.48)	1.99 (0.02 to 2.04)	2.89 (0.03 to 2.96)	3.94 (0.04 to 4.04)
24	1.47 (0.01 to 1.49)	2.01 (0.02 to 2.06)	2.92 (0.03 to 2.98)	3.97 (0.04 to 4.07)
25	1.49 (0.01 to 1.51)	2.03 (0.02 to 2.08)	2.95 (0.03 to 3.01)	4.01 (0.04 to 4.11)
26	1.51 (0.01 to 1.53)	2.06 (0.02 to 2.11)	2.99 (0.03 to 3.06)	4.07 (0.04 to 4.17)
27	1.54 (0.02 to 1.56)	2.10 (0.02 to 2.15)	3.05 (0.03 to 3.12)	4.15 (0.04 to 4.25)
28	1.59 (0.02 to 1.61)	2.17 (0.02 to 2.22)	3.15 (0.03 to 3.22)	4.28 (0.04 to 4.39)
29	1.66 (0.02 to 1.68)	2.27 (0.02 to 2.32)	3.29 (0.03 to 3.36)	4.47 (0.04 to 4.58)
30	1.75 (0.02 to 1.77)	2.39 (0.02 to 2.44)	3.46 (0.03 to 3.54)	4.70 (0.05 to 4.82)
31	1.84 (0.02 to 1.86)	2.51 (0.02 to 2.57)	3.64 (0.04 to 3.72)	4.95 (0.05 to 5.06)
32	1.94 (0.02 to 1.96)	2.65 (0.03 to 2.71)	3.84 (0.04 to 3.92)	5.21 (0.05 to 5.33)
33	2.06 (0.02 to 2.08)	2.81 (0.03 to 2.87)	4.07 (0.04 to 4.15)	5.52 (0.05 to 5.65)
34	2.20 (0.02 to 2.22)	2.99 (0.03 to 3.06)	4.34 (0.04 to 4.43)	5.88 (0.06 to 6.02)
35	2.36 (0.02 to 2.38)	3.21 (0.03 to 3.28)	4.64 (0.05 to 4.74)	6.29 (0.06 to 6.44)
36	2.53 (0.03 to 2.56)	3.45 (0.03 to 3.53)	4.99 (0.05 to 5.09)	6.76 (0.07 to 6.92)
37	2.73 (0.03 to 2.76)	3.71 (0.04 to 3.80)	5.37 (0.05 to 5.48)	7.27 (0.07 to 7.44)
38	2.95 (0.03 to 2.97)	4.01 (0.04 to 4.10)	5.79 (0.06 to 5.91)	7.83 (0.08 to 8.01)
39	3.18 (0.03 to 3.21)	4.32 (0.04 to 4.42)	6.24 (0.06 to 6.37)	8.43 (0.08 to 8.63)
40	3.43 (0.03 to 3.47)	4.66 (0.05 to 4.77)	6.72 (0.07 to 6.86)	9.07 (0.09 to 9.29)
41	3.70 (0.04 to 3.75)	5.02 (0.05 to 5.14)	7.24 (0.07 to 7.40)	9.76 (0.10 to 10.00)
42	3.99 (0.04 to 4.05)	5.41 (0.05 to 5.55)	7.79 (0.08 to 7.98)	10.50 (0.10 to 10.77)
43	4.30 (0.04 to 4.37)	5.83 (0.06 to 5.99)	8.39 (0.08 to 8.60)	11.29 (0.11 to 11.59)
44	4.64 (0.05 to 4.73)	6.29 (0.06 to 6.46)	9.03 (0.09 to 9.27)	12.14 (0.12 to 12.48)
45	5.00 (0.05 to 5.11)	6.77 (0.07 to 6.97)	9.72 (0.09 to 9.99)	13.05 (0.13 to 13.43)
46	5.39 (0.05 to 5.52)	7.30 (0.07 to 7.52)	10.46 (0.10 to 10.76)	14.02 (0.14 to 14.44)
47	5.81 (0.06 to 5.96)	7.86 (0.08 to 8.11)	11.25 (0.11 to 11.59)	15.05 (0.15 to 15.53)
48	6.26 (0.06 to 6.43)	8.46 (0.08 to 8.75)	12.09 (0.12 to 12.48)	16.16 (0.16 to 16.69)
49	6.74 (0.07 to 6.95)	9.11 (0.09 to 9.43)	13.00 (0.13 to 13.44)	17.33 (0.17 to 17.93)
**Eclampsia & preeclampsia**				
Maternal age (years)	nIVF_S	IVF_S	nIVF_T	IVF_T
20	2.28 (0.02 to 2.32)	2.87 (0.03 to 2.94)	7.22 (0.07 to 7.37)	9.03 (0.09 to 9.23)
21	2.19 (0.02 to 2.22)	2.75 (0.03 to 2.81)	6.93 (0.07 to 7.05)	8.67 (0.09 to 8.83)
22	2.10 (0.02 to 2.12)	2.64 (0.03 to 2.69)	6.65 (0.07 to 6.75)	8.32 (0.08 to 8.47)
23	2.02 (0.02 to 2.04)	2.54 (0.02 to 2.58)	6.40 (0.06 to 6.49)	8.01 (0.08 to 8.15)
24	1.95 (0.02 to 1.97)	2.45 (0.02 to 2.50)	6.19 (0.06 to 6.28)	7.75 (0.08 to 7.88)
25	1.89 (0.02 to 1.91)	2.39 (0.02 to 2.43)	6.02 (0.06 to 6.10)	7.54 (0.07 to 7.66)

26	1.86 (0.02 to 1.88)	2.35 (0.02 to 2.38)	5.92 (0.06 to 6.00)	7.42 (0.07 to 7.54)
27	1.87 (0.02 to 1.89)	2.35 (0.02 to 2.40)	5.95 (0.06 to 6.03)	7.45 (0.07 to 7.57)
28	1.93 (0.02 to 1.94)	2.43 (0.02 to 2.47)	6.13 (0.06 to 6.21)	7.67 (0.08 to 7.80)
29	2.01 (0.02 to 2.03)	2.53 (0.02 to 2.58)	6.39 (0.06 to 6.48)	8.00 (0.08 to 8.13)
30	2.09 (0.02 to 2.11)	2.64 (0.03 to 2.68)	6.64 (0.07 to 6.73)	8.31 (0.08 to 8.44)
31	2.20 (0.02 to 2.22)	2.77 (0.03 to 2.82)	6.97 (0.07 to 7.06)	8.71 (0.09 to 8.85)
32	2.37 (0.02 to 2.39)	2.98 (0.03 to 3.03)	7.48 (0.07 to 7.58)	9.35 (0.09 to 9.49)
33	2.58 (0.03 to 2.60)	3.24 (0.03 to 3.30)	8.13 (0.08 to 8.24)	10.16 (0.10 to 10.31)
34	2.81 (0.03 to 2.83)	3.54 (0.03 to 3.59)	8.84 (0.09 to 8.96)	11.03 (0.11 to 11.20)
35	3.06 (0.03 to 3.08)	3.84 (0.04 to 3.91)	9.59 (0.09 to 9.72)	11.95 (0.12 to 12.13)
36	3.32 (0.03 to 3.35)	4.17 (0.04 to 4.24)	10.38 (0.10 to 10.52)	12.92 (0.13 to 13.12)
37	3.60 (0.04 to 3.63)	4.52 (0.04 to 4.59)	11.22 (0.11 to 11.36)	13.94 (0.14 to 14.15)
38	3.89 (0.04 to 3.93)	4.89 (0.05 to 4.97)	12.10 (0.12 to 12.26)	15.02 (0.15 to 15.25)
39	4.21 (0.04 to 4.25)	5.29 (0.05 to 5.37)	13.04 (0.13 to 13.21)	16.17 (0.16 to 16.41)
40	4.55 (0.05 to 4.59)	5.71 (0.06 to 5.81)	14.05 (0.14 to 14.23)	17.39 (0.17 to 17.66)
41	4.92 (0.05 to 4.97)	6.17 (0.06 to 6.28)	15.12 (0.15 to 15.33)	18.69 (0.18 to 18.98)
42	5.32 (0.05 to 5.38)	6.67 (0.07 to 6.79)	16.27 (0.16 to 16.51)	20.08 (0.20 to 20.40)
43	5.75 (0.06 to 5.82)	7.20 (0.07 to 7.34)	17.49 (0.17 to 17.77)	21.55 (0.21 to 21.92)
44	6.21 (0.06 to 6.30)	7.78 (0.08 to 7.93)	18.80 (0.18 to 19.12)	23.11 (0.23 to 23.53)
45	6.71 (0.07 to 6.82)	8.39 (0.08 to 8.57)	20.19 (0.20 to 20.56)	24.77 (0.24 to 25.25)
46	7.25 (0.07 to 7.38)	9.06 (0.09 to 9.27)	21.67 (0.21 to 22.10)	26.53 (0.26 to 27.07)
47	7.82 (0.08 to 7.98)	9.77 (0.10 to 10.01)	23.24 (0.23 to 23.73)	28.39 (0.28 to 29.00)
48	8.45 (0.08 to 8.63)	10.54 (0.10 to 10.81)	24.91 (0.24 to 25.47)	30.35 (0.30 to 31.03)
49	9.11 (0.09 to 9.34)	11.36 (0.11 to 11.68)	26.68 (0.26 to 27.31)	32.41 (0.32 to 33.18)
**Gestational diabetes**				
Maternal age (years)	nIVF_S	IVF_S	nIVF_T	IVF_T
20	4.10 (0.04 to 4.17)	5.91 (0.06 to 6.02)	4.50 (0.04 to 4.58)	6.47 (0.06 to 6.59)
21	4.76 (0.05 to 4.82)	6.85 (0.07 to 6.95)	5.22 (0.05 to 5.30)	7.50 (0.07 to 7.61)
22	5.52 (0.05 to 5.57)	7.93 (0.08 to 8.02)	6.05 (0.06 to 6.12)	8.67 (0.09 to 8.79)
23	6.38 (0.06 to 6.42)	9.14 (0.09 to 9.24)	6.98 (0.07 to 7.06)	9.99 (0.10 to 10.11)
24	7.31 (0.07 to 7.36)	10.45 (0.10 to 10.56)	8.00 (0.08 to 8.09)	11.42 (0.11 to 11.56)
25	8.29 (0.08 to 8.35)	11.83 (0.12 to 11.95)	9.07 (0.09 to 9.17)	12.91 (0.13 to 13.07)
26	9.30 (0.09 to 9.35)	13.23 (0.13 to 13.36)	10.17 (0.10 to 10.28)	14.44 (0.14 to 14.60)
27	10.39 (0.10 to 10.45)	14.74 (0.15 to 14.89)	11.35 (0.11 to 11.47)	16.07 (0.16 to 16.25)
28	11.62 (0.12 to 11.69)	16.44 (0.16 to 16.60)	12.69 (0.13 to 12.82)	17.91 (0.18 to 18.11)
29	12.86 (0.13 to 12.93)	18.14 (0.18 to 18.31)	14.03 (0.14 to 14.18)	19.74 (0.20 to 19.95)
30	13.92 (0.14 to 13.99)	19.58 (0.19 to 19.76)	15.18 (0.15 to 15.33)	21.29 (0.21 to 21.51)
31	14.95 (0.15 to 15.02)	20.98 (0.21 to 21.16)	16.30 (0.16 to 16.46)	22.79 (0.23 to 23.02)
32	16.16 (0.16 to 16.23)	22.61 (0.22 to 22.80)	17.61 (0.17 to 17.77)	24.54 (0.24 to 24.78)
33	17.45 (0.17 to 17.49)	24.34 (0.24 to 24.53)	19.00 (0.19 to 19.17)	26.39 (0.26 to 26.63)
34	18.63 (0.19 to 18.69)	25.90 (0.26 to 26.09)	20.27 (0.20 to 20.44)	28.06 (0.28 to 28.30)
35	19.65 (0.20 to 19.71)	27.25 (0.27 to 27.43)	21.37 (0.21 to 21.53)	29.50 (0.29 to 29.73)

©2021 Wang Y et al. *JAMA Network Open*.

36	20.53 (0.20 to 20.59)	28.40 (0.28 to 28.56)	22.31 (0.22 to 22.46)	30.72 (0.30 to 30.94)
37	21.27 (0.21 to 21.34)	29.37 (0.29 to 29.50)	23.11 (0.23 to 23.24)	31.76 (0.32 to 31.96)
38	21.92 (0.22 to 21.99)	30.22 (0.30 to 30.28)	23.80 (0.24 to 23.91)	32.65 (0.32 to 32.82)
39	22.50 (0.22 to 22.57)	30.97 (0.31 to 31.00)	24.42 (0.24 to 24.48)	33.44 (0.33 to 33.58)
40	23.05 (0.23 to 23.05)	31.67 (0.32 to 31.70)	25.01 (0.25 to 25.02)	34.19 (0.34 to 34.28)
41	23.60 (0.24 to 23.60)	32.39 (0.32 to 32.42)	25.60 (0.26 to 25.61)	34.95 (0.35 to 34.96)
42	24.16 (0.24 to 24.17)	33.11 (0.33 to 33.14)	26.20 (0.26 to 26.21)	35.71 (0.36 to 35.73)
43	24.73 (0.25 to 24.74)	33.84 (0.34 to 33.88)	26.82 (0.27 to 26.82)	36.49 (0.36 to 36.50)
44	25.32 (0.25 to 25.33)	34.59 (0.35 to 34.62)	27.44 (0.27 to 27.45)	37.27 (0.37 to 37.29)
45	25.92 (0.26 to 25.92)	35.35 (0.35 to 35.38)	28.08 (0.28 to 28.08)	38.07 (0.38 to 38.09)
46	26.52 (0.27 to 26.53)	36.12 (0.36 to 36.15)	28.73 (0.29 to 28.73)	38.88 (0.39 to 38.90)
47	27.14 (0.27 to 27.15)	36.90 (0.37 to 36.93)	29.39 (0.29 to 29.39)	39.70 (0.40 to 39.77)
48	27.78 (0.28 to 27.78)	37.69 (0.38 to 37.73)	30.06 (0.30 to 30.10)	40.53 (0.40 to 40.67)
49	28.42 (0.28 to 28.43)	38.50 (0.38 to 38.58)	30.74 (0.31 to 30.85)	41.38 (0.41 to 41.56)
**Placenta previa**				
Maternal age (years)	nIVF_S	IVF_S	nIVF_T	IVF_T
20	0.50 (0.00 to 0.52)	0.91 (0.01 to 0.95)	0.48 (0.00 to 0.50)	0.87 (0.01 to 0.90)
21	0.55 (0.01 to 0.56)	1.00 (0.01 to 1.03)	0.52 (0.01 to 0.54)	0.95 (0.01 to 0.98)
22	0.60 (0.01 to 0.61)	1.09 (0.01 to 1.12)	0.57 (0.01 to 0.59)	1.04 (0.01 to 1.07)
23	0.66 (0.01 to 0.67)	1.20 (0.01 to 1.22)	0.63 (0.01 to 0.64)	1.14 (0.01 to 1.17)
24	0.72 (0.01 to 0.73)	1.31 (0.01 to 1.34)	0.69 (0.01 to 0.70)	1.24 (0.01 to 1.27)
25	0.79 (0.01 to 0.80)	1.42 (0.01 to 1.46)	0.75 (0.01 to 0.77)	1.35 (0.01 to 1.39)
26	0.86 (0.01 to 0.87)	1.56 (0.02 to 1.59)	0.82 (0.01 to 0.84)	1.48 (0.01 to 1.52)
27	0.95 (0.01 to 0.96)	1.72 (0.02 to 1.76)	0.90 (0.01 to 0.93)	1.63 (0.02 to 1.68)
28	1.07 (0.01 to 1.08)	1.94 (0.02 to 1.98)	1.02 (0.01 to 1.04)	1.84 (0.02 to 1.89)
29	1.22 (0.01 to 1.23)	2.20 (0.02 to 2.24)	1.16 (0.01 to 1.18)	2.09 (0.02 to 2.14)
30	1.36 (0.01 to 1.37)	2.45 (0.02 to 2.50)	1.29 (0.01 to 1.32)	2.33 (0.02 to 2.39)
31	1.51 (0.01 to 1.52)	2.72 (0.03 to 2.78)	1.43 (0.01 to 1.47)	2.58 (0.03 to 2.65)
32	1.68 (0.02 to 1.70)	3.03 (0.03 to 3.10)	1.60 (0.02 to 1.64)	2.88 (0.03 to 2.95)
33	1.87 (0.02 to 1.89)	3.37 (0.03 to 3.44)	1.78 (0.02 to 1.82)	3.21 (0.03 to 3.29)
34	2.06 (0.02 to 2.08)	3.71 (0.04 to 3.78)	1.96 (0.02 to 2.00)	3.52 (0.03 to 3.61)
35	2.23 (0.02 to 2.26)	4.02 (0.04 to 4.10)	2.12 (0.02 to 2.17)	3.82 (0.04 to 3.92)
36	2.40 (0.02 to 2.42)	4.31 (0.04 to 4.40)	2.28 (0.02 to 2.33)	4.10 (0.04 to 4.20)
37	2.55 (0.03 to 2.58)	4.58 (0.04 to 4.68)	2.42 (0.02 to 2.48)	4.36 (0.04 to 4.47)
38	2.70 (0.03 to 2.72)	4.84 (0.05 to 4.94)	2.56 (0.03 to 2.62)	4.60 (0.04 to 4.72)
39	2.84 (0.03 to 2.87)	5.09 (0.05 to 5.20)	2.70 (0.03 to 2.76)	4.84 (0.05 to 4.96)
40	2.98 (0.03 to 3.01)	5.34 (0.05 to 5.46)	2.83 (0.03 to 2.90)	5.08 (0.05 to 5.21)
41	3.13 (0.03 to 3.17)	5.60 (0.05 to 5.73)	2.97 (0.03 to 3.05)	5.33 (0.05 to 5.47)
42	3.28 (0.03 to 3.33)	5.88 (0.06 to 6.01)	3.12 (0.03 to 3.20)	5.59 (0.05 to 5.74)
43	3.45 (0.03 to 3.50)	6.17 (0.06 to 6.31)	3.27 (0.03 to 3.36)	5.86 (0.06 to 6.03)
44	3.62 (0.04 to 3.68)	6.47 (0.06 to 6.63)	3.44 (0.03 to 3.54)	6.15 (0.06 to 6.33)
45	3.79 (0.04 to 3.87)	6.78 (0.07 to 6.96)	3.61 (0.03 to 3.72)	6.45 (0.06 to 6.65)

©2021 Wang Y et al. *JAMA Network Open*.

46	3.98 (0.04 to 4.07)	7.11 (0.07 to 7.32)	3.78 (0.04 to 3.91)	6.76 (0.07 to 6.98)
47	4.18 (0.04 to 4.29)	7.45 (0.07 to 7.68)	3.97 (0.04 to 4.11)	7.09 (0.07 to 7.33)
48	4.38 (0.04 to 4.51)	7.81 (0.08 to 8.07)	4.17 (0.04 to 4.32)	7.44 (0.07 to 7.70)
49	4.60 (0.04 to 4.74)	8.19 (0.08 to 8.48)	4.37 (0.04 to 4.54)	7.79 (0.08 to 8.09)
**Placental abruption**				
Maternal age (years)	nIVF_S	IVF_S	nIVF_T	IVF_T
20	0.76 (0.01 to 0.78)	0.84 (0.01 to 0.88)	1.10 (0.01 to 1.16)	1.22 (0.01 to 1.29)
21	0.71 (0.01 to 0.73)	0.79 (0.01 to 0.83)	1.04 (0.01 to 1.08)	1.15 (0.01 to 1.21)
22	0.68 (0.01 to 0.69)	0.75 (0.01 to 0.78)	0.98 (0.01 to 1.02)	1.09 (0.01 to 1.14)
23	0.64 (0.01 to 0.65)	0.71 (0.01 to 0.74)	0.94 (0.01 to 0.97)	1.04 (0.01 to 1.08)
24	0.62 (0.01 to 0.63)	0.69 (0.01 to 0.72)	0.90 (0.01 to 0.94)	1.00 (0.01 to 1.05)
25	0.61 (0.01 to 0.62)	0.68 (0.01 to 0.71)	0.89 (0.01 to 0.93)	0.99 (0.01 to 1.03)
26	0.62 (0.01 to 0.63)	0.69 (0.01 to 0.72)	0.91 (0.01 to 0.94)	1.00 (0.01 to 1.05)
27	0.64 (0.01 to 0.65)	0.71 (0.01 to 0.74)	0.94 (0.01 to 0.97)	1.04 (0.01 to 1.08)
28	0.67 (0.01 to 0.68)	0.74 (0.01 to 0.77)	0.98 (0.01 to 1.01)	1.08 (0.01 to 1.13)
29	0.70 (0.01 to 0.71)	0.77 (0.01 to 0.80)	1.01 (0.01 to 1.05)	1.12 (0.01 to 1.17)
30	0.71 (0.01 to 0.72)	0.79 (0.01 to 0.82)	1.03 (0.01 to 1.07)	1.14 (0.01 to 1.19)
31	0.72 (0.01 to 0.74)	0.80 (0.01 to 0.84)	1.06 (0.01 to 1.09)	1.17 (0.01 to 1.22)
32	0.75 (0.01 to 0.77)	0.83 (0.01 to 0.87)	1.10 (0.01 to 1.14)	1.21 (0.01 to 1.27)
33	0.79 (0.01 to 0.80)	0.87 (0.01 to 0.91)	1.15 (0.01 to 1.19)	1.27 (0.01 to 1.33)
34	0.83 (0.01 to 0.84)	0.91 (0.01 to 0.95)	1.20 (0.01 to 1.25)	1.33 (0.01 to 1.39)
35	0.86 (0.01 to 0.87)	0.95 (0.01 to 0.99)	1.25 (0.01 to 1.30)	1.39 (0.01 to 1.45)
36	0.89 (0.01 to 0.91)	0.99 (0.01 to 1.03)	1.30 (0.01 to 1.35)	1.44 (0.01 to 1.50)
37	0.93 (0.01 to 0.94)	1.02 (0.01 to 1.07)	1.35 (0.01 to 1.40)	1.49 (0.01 to 1.55)
38	0.96 (0.01 to 0.97)	1.06 (0.01 to 1.10)	1.39 (0.01 to 1.44)	1.54 (0.01 to 1.61)
39	0.99 (0.01 to 1.00)	1.09 (0.01 to 1.14)	1.43 (0.01 to 1.49)	1.59 (0.02 to 1.66)
40	1.02 (0.01 to 1.04)	1.13 (0.01 to 1.17)	1.48 (0.01 to 1.54)	1.64 (0.02 to 1.71)
41	1.05 (0.01 to 1.07)	1.16 (0.01 to 1.21)	1.52 (0.01 to 1.59)	1.69 (0.02 to 1.77)
42	1.08 (0.01 to 1.11)	1.20 (0.01 to 1.25)	1.57 (0.02 to 1.64)	1.74 (0.02 to 1.82)
43	1.11 (0.01 to 1.15)	1.23 (0.01 to 1.29)	1.62 (0.02 to 1.69)	1.79 (0.02 to 1.89)
44	1.15 (0.01 to 1.19)	1.27 (0.01 to 1.34)	1.67 (0.02 to 1.75)	1.85 (0.02 to 1.95)
45	1.18 (0.01 to 1.23)	1.31 (0.01 to 1.38)	1.72 (0.02 to 1.81)	1.90 (0.02 to 2.02)
46	1.22 (0.01 to 1.28)	1.35 (0.01 to 1.43)	1.77 (0.02 to 1.88)	1.96 (0.02 to 2.09)
47	1.26 (0.01 to 1.32)	1.39 (0.01 to 1.48)	1.83 (0.02 to 1.94)	2.02 (0.02 to 2.16)
48	1.30 (0.01 to 1.37)	1.43 (0.01 to 1.53)	1.89 (0.02 to 2.01)	2.09 (0.02 to 2.23)
49	1.34 (0.01 to 1.42)	1.48 (0.01 to 1.59)	1.94 (0.02 to 2.08)	2.15 (0.02 to 2.31)
**Placenta accreta**				
Maternal age (years)	nIVF_S	IVF_S	nIVF_T	IVF_T
20	0.94 (0.01 to 0.96)	1.73 (0.02 to 1.79)	1.20 (0.01 to 1.23)	2.20 (0.02 to 2.27)
21	0.95 (0.01 to 0.97)	1.76 (0.02 to 1.80)	1.21 (0.01 to 1.25)	2.23 (0.02 to 2.30)
22	0.97 (0.01 to 0.98)	1.78 (0.02 to 1.82)	1.23 (0.01 to 1.26)	2.27 (0.02 to 2.32)

©2021 Wang Y et al. *JAMA Network Open*.

23	0.98 (0.01 to 1.00)	1.81 (0.02 to 1.85)	1.25 (0.01 to 1.28)	2.30 (0.02 to 2.36)
24	1.00 (0.01 to 1.02)	1.85 (0.02 to 1.89)	1.28 (0.01 to 1.31)	2.35 (0.02 to 2.41)
25	1.03 (0.01 to 1.04)	1.89 (0.02 to 1.93)	1.31 (0.01 to 1.34)	2.40 (0.02 to 2.46)
26	1.06 (0.01 to 1.07)	1.95 (0.02 to 1.99)	1.35 (0.01 to 1.38)	2.48 (0.02 to 2.54)
27	1.11 (0.01 to 1.12)	2.05 (0.02 to 2.09)	1.41 (0.01 to 1.45)	2.60 (0.03 to 2.66)
28	1.19 (0.01 to 1.20)	2.20 (0.02 to 2.24)	1.52 (0.01 to 1.55)	2.79 (0.03 to 2.85)
29	1.29 (0.01 to 1.30)	2.37 (0.02 to 2.42)	1.64 (0.02 to 1.68)	3.01 (0.03 to 3.08)
30	1.38 (0.01 to 1.39)	2.54 (0.02 to 2.59)	1.76 (0.02 to 1.80)	3.22 (0.03 to 3.30)
31	1.47 (0.01 to 1.49)	2.71 (0.03 to 2.77)	1.87 (0.02 to 1.92)	3.44 (0.03 to 3.52)
32	1.58 (0.02 to 1.59)	2.89 (0.03 to 2.95)	2.00 (0.02 to 2.05)	3.67 (0.04 to 3.76)
33	1.68 (0.02 to 1.69)	3.08 (0.03 to 3.15)	2.13 (0.02 to 2.18)	3.91 (0.04 to 4.00)
34	1.78 (0.02 to 1.80)	3.27 (0.03 to 3.34)	2.26 (0.02 to 2.31)	4.15 (0.04 to 4.24)
35	1.88 (0.02 to 1.90)	3.45 (0.03 to 3.52)	2.39 (0.02 to 2.44)	4.37 (0.04 to 4.47)
36	1.97 (0.02 to 1.99)	3.61 (0.04 to 3.69)	2.50 (0.02 to 2.56)	4.58 (0.04 to 4.68)
37	2.06 (0.02 to 2.08)	3.77 (0.04 to 3.85)	2.61 (0.03 to 2.67)	4.78 (0.05 to 4.89)
38	2.14 (0.02 to 2.17)	3.93 (0.04 to 4.01)	2.72 (0.03 to 2.78)	4.98 (0.05 to 5.09)
39	2.23 (0.02 to 2.25)	4.08 (0.04 to 4.17)	2.83 (0.03 to 2.89)	5.17 (0.05 to 5.29)
40	2.31 (0.02 to 2.34)	4.24 (0.04 to 4.33)	2.94 (0.03 to 3.01)	5.37 (0.05 to 5.49)
41	2.40 (0.02 to 2.44)	4.40 (0.04 to 4.50)	3.05 (0.03 to 3.12)	5.57 (0.05 to 5.71)
42	2.50 (0.02 to 2.53)	4.57 (0.04 to 4.67)	3.17 (0.03 to 3.25)	5.78 (0.06 to 5.93)
43	2.59 (0.03 to 2.64)	4.74 (0.05 to 4.86)	3.29 (0.03 to 3.38)	6.00 (0.06 to 6.16)
44	2.69 (0.03 to 2.75)	4.92 (0.05 to 5.05)	3.41 (0.03 to 3.51)	6.22 (0.06 to 6.40)
45	2.79 (0.03 to 2.86)	5.11 (0.05 to 5.25)	3.54 (0.03 to 3.65)	6.46 (0.06 to 6.66)
46	2.90 (0.03 to 2.98)	5.30 (0.05 to 5.46)	3.68 (0.04 to 3.80)	6.70 (0.06 to 6.92)
47	3.01 (0.03 to 3.10)	5.50 (0.05 to 5.68)	3.82 (0.04 to 3.95)	6.95 (0.07 to 7.19)
48	3.13 (0.03 to 3.23)	5.71 (0.06 to 5.91)	3.97 (0.04 to 4.11)	7.21 (0.07 to 7.48)
49	3.25 (0.03 to 3.36)	5.92 (0.06 to 6.15)	4.12 (0.04 to 4.28)	7.48 (0.07 to 7.78)
**Preterm birth**				
Maternal age (years)	nIVF_S	IVF_S	nIVF_T	IVF_T
20	5.62 (0.06 to 5.69)	6.58 (0.06 to 6.68)	31.98 (0.32 to 32.36)	36.46 (0.36 to 36.92)
21	5.22 (0.05 to 5.27)	6.12 (0.06 to 6.20)	30.04 (0.30 to 30.33)	34.32 (0.34 to 34.69)
22	4.85 (0.05 to 4.89)	5.69 (0.06 to 5.76)	28.22 (0.28 to 28.45)	32.30 (0.32 to 32.62)
23	4.54 (0.05 to 4.57)	5.32 (0.05 to 5.38)	26.61 (0.26 to 26.83)	30.51 (0.30 to 30.81)
24	4.29 (0.04 to 4.32)	5.03 (0.05 to 5.09)	25.33 (0.25 to 25.54)	29.08 (0.29 to 29.37)
25	4.12 (0.04 to 4.15)	4.83 (0.05 to 4.89)	24.46 (0.24 to 24.66)	28.11 (0.28 to 28.38)
26	4.05 (0.04 to 4.07)	4.75 (0.05 to 4.80)	24.06 (0.24 to 24.25)	27.67 (0.27 to 27.93)
27	4.05 (0.04 to 4.07)	4.75 (0.05 to 4.80)	24.06 (0.24 to 24.26)	27.67 (0.27 to 27.93)
28	4.11 (0.04 to 4.13)	4.82 (0.05 to 4.87)	24.38 (0.24 to 24.56)	28.02 (0.28 to 28.28)
29	4.22 (0.04 to 4.24)	4.94 (0.05 to 5.00)	24.95 (0.25 to 25.15)	28.66 (0.28 to 28.93)
30	4.37 (0.04 to 4.39)	5.12 (0.05 to 5.17)	25.73 (0.26 to 25.92)	29.53 (0.29 to 29.80)
31	4.56 (0.05 to 4.58)	5.34 (0.05 to 5.40)	26.71 (0.26 to 26.92)	30.62 (0.30 to 30.90)
32	4.78 (0.05 to 4.81)	5.61 (0.06 to 5.67)	27.87 (0.28 to 28.08)	31.91 (0.32 to 32.20)

©2021 Wang Y et al. *JAMA Network Open*.

33	5.03 (0.05 to 5.06)	5.89 (0.06 to 5.96)	29.10 (0.29 to 29.31)	33.28 (0.33 to 33.57)
34	5.27 (0.05 to 5.30)	6.18 (0.06 to 6.24)	30.30 (0.30 to 30.52)	34.61 (0.34 to 34.90)
35	5.51 (0.05 to 5.54)	6.45 (0.06 to 6.52)	31.44 (0.31 to 31.67)	35.86 (0.36 to 36.17)
36	5.74 (0.06 to 5.77)	6.71 (0.07 to 6.79)	32.53 (0.32 to 32.77)	37.06 (0.37 to 37.38)
37	5.96 (0.06 to 5.99)	6.97 (0.07 to 7.05)	33.58 (0.33 to 33.83)	38.21 (0.38 to 38.53)
38	6.18 (0.06 to 6.21)	7.23 (0.07 to 7.31)	34.60 (0.34 to 34.85)	39.33 (0.39 to 39.66)
39	6.39 (0.06 to 6.43)	7.48 (0.07 to 7.56)	35.61 (0.35 to 35.87)	40.43 (0.40 to 40.77)
40	6.62 (0.07 to 6.66)	7.74 (0.08 to 7.83)	36.62 (0.36 to 36.91)	41.53 (0.41 to 41.89)
41	6.84 (0.07 to 6.90)	8.00 (0.08 to 8.10)	37.65 (0.37 to 37.97)	42.64 (0.42 to 43.04)
42	7.08 (0.07 to 7.15)	8.28 (0.08 to 8.39)	38.69 (0.38 to 39.06)	43.77 (0.43 to 44.21)
43	7.33 (0.07 to 7.41)	8.56 (0.08 to 8.69)	39.76 (0.39 to 40.17)	44.92 (0.44 to 45.41)
44	7.58 (0.07 to 7.68)	8.86 (0.09 to 9.00)	40.84 (0.40 to 41.31)	46.08 (0.46 to 46.62)
45	7.84 (0.08 to 7.95)	9.16 (0.09 to 9.32)	41.95 (0.41 to 42.48)	47.27 (0.47 to 47.86)
46	8.11 (0.08 to 8.24)	9.47 (0.09 to 9.65)	43.07 (0.42 to 43.66)	48.46 (0.48 to 49.12)
47	8.39 (0.08 to 8.54)	9.79 (0.10 to 9.99)	44.21 (0.44 to 44.86)	49.67 (0.49 to 50.40)
48	8.67 (0.09 to 8.85)	10.13 (0.10 to 10.35)	45.36 (0.45 to 46.09)	50.89 (0.50 to 51.69)
49	8.97 (0.09 to 9.17)	10.47 (0.10 to 10.72)	46.53 (0.46 to 47.33)	52.13 (0.51 to 53.00)
**Dystocia**				
Maternal age (years)	nIVF_S	IVF_S	nIVF_T	IVF_T
20	6.46 (0.06 to 6.53)	8.50 (0.08 to 8.63)	9.41 (0.09 to 9.54)	12.32 (0.12 to 12.51)
21	6.63 (0.07 to 6.68)	8.71 (0.09 to 8.83)	9.65 (0.10 to 9.76)	12.62 (0.12 to 12.79)
22	6.80 (0.07 to 6.83)	8.93 (0.09 to 9.04)	9.89 (0.10 to 9.99)	12.93 (0.13 to 13.09)
23	6.97 (0.07 to 7.01)	9.16 (0.09 to 9.27)	10.14 (0.10 to 10.24)	13.25 (0.13 to 13.41)
24	7.15 (0.07 to 7.19)	9.40 (0.09 to 9.51)	10.40 (0.10 to 10.51)	13.59 (0.13 to 13.76)
25	7.35 (0.07 to 7.39)	9.65 (0.10 to 9.77)	10.68 (0.11 to 10.79)	13.95 (0.14 to 14.12)
26	7.55 (0.08 to 7.59)	9.92 (0.10 to 10.03)	10.97 (0.11 to 11.08)	14.32 (0.14 to 14.49)
27	7.72 (0.08 to 7.76)	10.13 (0.10 to 10.25)	11.21 (0.11 to 11.32)	14.62 (0.14 to 14.80)
28	7.80 (0.08 to 7.84)	10.24 (0.10 to 10.36)	11.33 (0.11 to 11.44)	14.77 (0.15 to 14.95)
29	7.79 (0.08 to 7.83)	10.22 (0.10 to 10.34)	11.31 (0.11 to 11.42)	14.75 (0.15 to 14.92)
30	7.69 (0.08 to 7.72)	10.09 (0.10 to 10.21)	11.16 (0.11 to 11.28)	14.56 (0.14 to 14.74)
31	7.53 (0.07 to 7.57)	9.89 (0.10 to 10.01)	10.94 (0.11 to 11.06)	14.28 (0.14 to 14.45)
32	7.37 (0.07 to 7.40)	9.68 (0.10 to 9.79)	10.71 (0.11 to 10.82)	13.98 (0.14 to 14.15)
33	7.23 (0.07 to 7.27)	9.50 (0.09 to 9.61)	10.52 (0.10 to 10.62)	13.74 (0.14 to 13.90)
34	7.16 (0.07 to 7.20)	9.41 (0.09 to 9.52)	10.41 (0.10 to 10.52)	13.60 (0.13 to 13.76)
35	7.15 (0.07 to 7.18)	9.39 (0.09 to 9.50)	10.39 (0.10 to 10.50)	13.57 (0.13 to 13.74)
36	7.18 (0.07 to 7.22)	9.43 (0.09 to 9.54)	10.44 (0.10 to 10.55)	13.64 (0.13 to 13.80)
37	7.25 (0.07 to 7.29)	9.53 (0.09 to 9.64)	10.54 (0.10 to 10.65)	13.77 (0.14 to 13.93)
38	7.36 (0.07 to 7.39)	9.66 (0.10 to 9.77)	10.69 (0.11 to 10.80)	13.96 (0.14 to 14.13)
39	7.48 (0.07 to 7.52)	9.82 (0.10 to 9.94)	10.87 (0.11 to 10.99)	14.19 (0.14 to 14.36)
40	7.62 (0.08 to 7.67)	10.00 (0.10 to 10.12)	11.06 (0.11 to 11.19)	14.44 (0.14 to 14.62)
41	7.76 (0.08 to 7.82)	10.18 (0.10 to 10.32)	11.27 (0.11 to 11.40)	14.69 (0.15 to 14.89)
42	7.90 (0.08 to 7.98)	10.37 (0.10 to 10.51)	11.47 (0.11 to 11.62)	14.96 (0.15 to 15.17)

©2021 Wang Y et al. *JAMA Network Open*.

43	8.05 (0.08 to 8.14)	10.56 (0.10 to 10.72)	11.68 (0.12 to 11.84)	15.22 (0.15 to 15.45)
44	8.20 (0.08 to 8.30)	10.75 (0.11 to 10.93)	11.89 (0.12 to 12.07)	15.49 (0.15 to 15.74)
45	8.35 (0.08 to 8.47)	10.95 (0.11 to 11.14)	12.11 (0.12 to 12.31)	15.77 (0.15 to 16.04)
46	8.51 (0.08 to 8.64)	11.15 (0.11 to 11.36)	12.33 (0.12 to 12.55)	16.05 (0.16 to 16.35)
47	8.66 (0.09 to 8.82)	11.35 (0.11 to 11.58)	12.55 (0.12 to 12.80)	16.33 (0.16 to 16.66)
48	8.82 (0.09 to 9.00)	11.56 (0.11 to 11.81)	12.78 (0.13 to 13.05)	16.62 (0.16 to 16.98)
49	8.99 (0.09 to 9.18)	11.77 (0.12 to 12.04)	13.01 (0.13 to 13.30)	16.91 (0.17 to 17.30)
**Cesarean section**				
Maternal age (years)	nIVF_S	IVF_S	nIVF_T	IVF_T
20	22.35 (0.22 to 22.46)	26.38 (0.26 to 26.54)	34.29 (0.34 to 34.47)	39.85 (0.40 to 40.08)
21	23.25 (0.23 to 23.33)	27.41 (0.27 to 27.55)	35.54 (0.35 to 35.70)	41.24 (0.41 to 41.44)
22	24.17 (0.24 to 24.23)	28.46 (0.28 to 28.60)	36.83 (0.37 to 36.97)	42.65 (0.42 to 42.83)
23	25.10 (0.25 to 25.16)	29.53 (0.29 to 29.66)	38.11 (0.38 to 38.25)	44.06 (0.44 to 44.24)
24	26.02 (0.26 to 26.07)	30.57 (0.30 to 30.70)	39.36 (0.39 to 39.50)	45.42 (0.45 to 45.61)
25	26.89 (0.27 to 26.94)	31.56 (0.31 to 31.69)	40.54 (0.40 to 40.68)	46.71 (0.47 to 46.89)
26	27.72 (0.28 to 27.77)	32.50 (0.32 to 32.64)	41.66 (0.42 to 41.81)	47.92 (0.48 to 48.11)
27	28.68 (0.29 to 28.73)	33.58 (0.33 to 33.72)	42.94 (0.43 to 43.08)	49.29 (0.49 to 49.48)
28	29.90 (0.30 to 29.95)	34.95 (0.35 to 35.09)	44.55 (0.44 to 44.69)	51.02 (0.51 to 51.21)
29	31.27 (0.31 to 31.33)	36.49 (0.36 to 36.64)	46.34 (0.46 to 46.49)	52.93 (0.53 to 53.13)
30	32.65 (0.33 to 32.70)	38.03 (0.38 to 38.18)	48.12 (0.48 to 48.27)	54.81 (0.55 to 55.00)
31	34.08 (0.34 to 34.14)	39.62 (0.39 to 39.77)	49.93 (0.50 to 50.08)	56.71 (0.57 to 56.91)
32	35.60 (0.36 to 35.66)	41.30 (0.41 to 41.46)	51.83 (0.52 to 51.99)	58.70 (0.59 to 58.90)
33	37.07 (0.37 to 37.12)	42.91 (0.43 to 43.07)	53.64 (0.53 to 53.79)	60.57 (0.60 to 60.76)
34	38.29 (0.38 to 38.35)	44.25 (0.44 to 44.42)	55.12 (0.55 to 55.28)	62.09 (0.62 to 62.29)
35	39.26 (0.39 to 39.32)	45.31 (0.45 to 45.48)	56.29 (0.56 to 56.45)	63.28 (0.63 to 63.47)
36	40.00 (0.40 to 40.07)	46.12 (0.46 to 46.29)	57.17 (0.57 to 57.34)	64.17 (0.64 to 64.37)
37	40.56 (0.40 to 40.63)	46.73 (0.47 to 46.90)	57.83 (0.58 to 58.00)	64.84 (0.65 to 65.04)
38	40.98 (0.41 to 41.05)	47.18 (0.47 to 47.35)	58.32 (0.58 to 58.49)	65.34 (0.65 to 65.53)
39	41.30 (0.41 to 41.37)	47.53 (0.47 to 47.71)	58.70 (0.59 to 58.87)	65.72 (0.66 to 65.92)
40	41.58 (0.41 to 41.66)	47.83 (0.48 to 48.01)	59.02 (0.59 to 59.20)	66.04 (0.66 to 66.25)
41	41.85 (0.42 to 41.95)	48.12 (0.48 to 48.31)	59.34 (0.59 to 59.52)	66.36 (0.66 to 66.57)
42	42.12 (0.42 to 42.24)	48.41 (0.48 to 48.62)	59.65 (0.59 to 59.85)	66.67 (0.66 to 66.90)
43	42.39 (0.42 to 42.53)	48.71 (0.48 to 48.92)	59.97 (0.60 to 60.18)	66.98 (0.67 to 67.22)
44	42.66 (0.43 to 42.82)	49.00 (0.49 to 49.23)	60.28 (0.60 to 60.51)	67.30 (0.67 to 67.55)
45	42.94 (0.43 to 43.12)	49.30 (0.49 to 49.55)	60.59 (0.60 to 60.85)	67.61 (0.67 to 67.88)
46	43.21 (0.43 to 43.42)	49.59 (0.49 to 49.86)	60.91 (0.61 to 61.18)	67.92 (0.68 to 68.22)
47	43.49 (0.43 to 43.71)	49.89 (0.50 to 50.18)	61.22 (0.61 to 61.52)	68.24 (0.68 to 68.55)
48	43.76 (0.44 to 44.01)	50.18 (0.50 to 50.50)	61.54 (0.61 to 61.86)	68.55 (0.68 to 68.88)
49	44.04 (0.44 to 44.32)	50.48 (0.50 to 50.82)	61.85 (0.62 to 62.20)	68.86 (0.69 to 69.21)
**Postpartum hemorrhage**				
Maternal age (years)	nIVF_S	IVF_S	nIVF_T	IVF_T

©2021 Wang Y et al. *JAMA Network Open*.

20	3.17 (0.03 to 3.22)	5.13 (0.05 to 5.23)	5.46 (0.05 to 5.57)	8.77 (0.09 to 8.95)
21	3.17 (0.03 to 3.20)	5.12 (0.05 to 5.21)	5.46 (0.05 to 5.55)	8.76 (0.09 to 8.91)
22	3.16 (0.03 to 3.19)	5.12 (0.05 to 5.20)	5.45 (0.05 to 5.53)	8.75 (0.09 to 8.89)
23	3.17 (0.03 to 3.19)	5.13 (0.05 to 5.21)	5.46 (0.05 to 5.54)	8.76 (0.09 to 8.90)
24	3.19 (0.03 to 3.21)	5.16 (0.05 to 5.24)	5.49 (0.05 to 5.57)	8.82 (0.09 to 8.96)
25	3.23 (0.03 to 3.26)	5.23 (0.05 to 5.31)	5.57 (0.05 to 5.65)	8.93 (0.09 to 9.07)
26	3.31 (0.03 to 3.33)	5.35 (0.05 to 5.43)	5.69 (0.06 to 5.77)	9.13 (0.09 to 9.27)
27	3.42 (0.03 to 3.44)	5.53 (0.05 to 5.61)	5.89 (0.06 to 5.97)	9.43 (0.09 to 9.58)
28	3.57 (0.04 to 3.59)	5.76 (0.06 to 5.85)	6.14 (0.06 to 6.22)	9.83 (0.10 to 9.98)
29	3.71 (0.04 to 3.73)	5.99 (0.06 to 6.08)	6.37 (0.06 to 6.46)	10.20 (0.10 to 10.36)
30	3.80 (0.04 to 3.82)	6.13 (0.06 to 6.22)	6.53 (0.06 to 6.62)	10.44 (0.10 to 10.60)
31	3.87 (0.04 to 3.90)	6.25 (0.06 to 6.34)	6.65 (0.07 to 6.75)	10.64 (0.10 to 10.80)
32	3.98 (0.04 to 4.00)	6.41 (0.06 to 6.51)	6.83 (0.07 to 6.92)	10.91 (0.11 to 11.07)
33	4.10 (0.04 to 4.12)	6.61 (0.07 to 6.70)	7.03 (0.07 to 7.13)	11.23 (0.11 to 11.39)
34	4.22 (0.04 to 4.24)	6.80 (0.07 to 6.89)	7.23 (0.07 to 7.33)	11.54 (0.11 to 11.71)
35	4.33 (0.04 to 4.36)	6.98 (0.07 to 7.08)	7.43 (0.07 to 7.53)	11.84 (0.12 to 12.02)
36	4.44 (0.04 to 4.47)	7.15 (0.07 to 7.25)	7.61 (0.08 to 7.71)	12.13 (0.12 to 12.31)
37	4.54 (0.05 to 4.57)	7.31 (0.07 to 7.42)	7.78 (0.08 to 7.89)	12.40 (0.12 to 12.58)
38	4.64 (0.05 to 4.68)	7.47 (0.07 to 7.58)	7.95 (0.08 to 8.06)	12.66 (0.12 to 12.85)
39	4.74 (0.05 to 4.78)	7.63 (0.08 to 7.75)	8.12 (0.08 to 8.24)	12.93 (0.13 to 13.12)
40	4.85 (0.05 to 4.89)	7.79 (0.08 to 7.91)	8.29 (0.08 to 8.42)	13.19 (0.13 to 13.39)
41	4.95 (0.05 to 5.00)	7.96 (0.08 to 8.08)	8.47 (0.08 to 8.60)	13.45 (0.13 to 13.67)
42	5.05 (0.05 to 5.11)	8.12 (0.08 to 8.26)	8.64 (0.08 to 8.79)	13.73 (0.13 to 13.97)
43	5.16 (0.05 to 5.23)	8.29 (0.08 to 8.45)	8.82 (0.09 to 8.98)	14.00 (0.14 to 14.26)
44	5.27 (0.05 to 5.35)	8.46 (0.08 to 8.63)	9.00 (0.09 to 9.18)	14.28 (0.14 to 14.57)
45	5.38 (0.05 to 5.48)	8.64 (0.08 to 8.83)	9.19 (0.09 to 9.39)	14.57 (0.14 to 14.89)
46	5.49 (0.05 to 5.61)	8.82 (0.09 to 9.03)	9.38 (0.09 to 9.60)	14.86 (0.15 to 15.21)
47	5.61 (0.05 to 5.74)	9.00 (0.09 to 9.23)	9.57 (0.09 to 9.82)	15.16 (0.15 to 15.54)
48	5.73 (0.06 to 5.87)	9.19 (0.09 to 9.44)	9.77 (0.10 to 10.04)	15.46 (0.15 to 15.88)
49	5.85 (0.06 to 6.01)	9.38 (0.09 to 9.66)	9.97 (0.10 to 10.27)	15.77 (0.15 to 16.23)
**Fetal growth restriction**				
Maternal age (years)	nIVF_S	IVF_S	nIVF_T	IVF_T
20	1.31 (0.01 to 1.34)	1.42 (0.01 to 1.48)	7.51 (0.07 to 7.74)	8.14 (0.08 to 8.44)
21	1.20 (0.01 to 1.23)	1.31 (0.01 to 1.35)	6.93 (0.07 to 7.12)	7.52 (0.07 to 7.76)
22	1.11 (0.01 to 1.13)	1.20 (0.01 to 1.24)	6.40 (0.06 to 6.55)	6.94 (0.07 to 7.15)
23	1.02 (0.01 to 1.04)	1.11 (0.01 to 1.14)	5.91 (0.06 to 6.04)	6.41 (0.06 to 6.60)
24	0.95 (0.01 to 0.96)	1.03 (0.01 to 1.06)	5.49 (0.05 to 5.61)	5.96 (0.06 to 6.13)
25	0.89 (0.01 to 0.90)	0.97 (0.01 to 1.00)	5.15 (0.05 to 5.27)	5.60 (0.05 to 5.76)
26	0.85 (0.01 to 0.86)	0.92 (0.01 to 0.95)	4.92 (0.05 to 5.03)	5.35 (0.05 to 5.50)
27	0.83 (0.01 to 0.84)	0.90 (0.01 to 0.93)	4.80 (0.05 to 4.91)	5.21 (0.05 to 5.36)
28	0.82 (0.01 to 0.83)	0.89 (0.01 to 0.92)	4.78 (0.05 to 4.88)	5.19 (0.05 to 5.34)
29	0.83 (0.01 to 0.84)	0.90 (0.01 to 0.93)	4.82 (0.05 to 4.93)	5.24 (0.05 to 5.38)

©2021 Wang Y et al. *JAMA Network Open*.

30	0.84 (0.01 to 0.85)	0.91 (0.01 to 0.94)	4.89 (0.05 to 5.00)	5.31 (0.05 to 5.46)
31	0.85 (0.01 to 0.86)	0.92 (0.01 to 0.95)	4.92 (0.05 to 5.02)	5.34 (0.05 to 5.49)
32	0.85 (0.01 to 0.86)	0.93 (0.01 to 0.95)	4.95 (0.05 to 5.05)	5.37 (0.05 to 5.52)
33	0.86 (0.01 to 0.88)	0.94 (0.01 to 0.97)	5.02 (0.05 to 5.13)	5.45 (0.05 to 5.60)
34	0.88 (0.01 to 0.90)	0.96 (0.01 to 0.99)	5.14 (0.05 to 5.25)	5.58 (0.05 to 5.74)
35	0.91 (0.01 to 0.93)	0.99 (0.01 to 1.02)	5.30 (0.05 to 5.42)	5.76 (0.06 to 5.91)
36	0.95 (0.01 to 0.96)	1.03 (0.01 to 1.06)	5.49 (0.05 to 5.62)	5.97 (0.06 to 6.13)
37	0.99 (0.01 to 1.00)	1.07 (0.01 to 1.10)	5.71 (0.06 to 5.84)	6.20 (0.06 to 6.37)
38	1.03 (0.01 to 1.04)	1.12 (0.01 to 1.15)	5.95 (0.06 to 6.09)	6.46 (0.06 to 6.64)
39	1.07 (0.01 to 1.09)	1.17 (0.01 to 1.20)	6.20 (0.06 to 6.35)	6.74 (0.07 to 6.93)
40	1.12 (0.01 to 1.14)	1.22 (0.01 to 1.26)	6.47 (0.06 to 6.63)	7.02 (0.07 to 7.23)
41	1.17 (0.01 to 1.19)	1.27 (0.01 to 1.31)	6.74 (0.07 to 6.92)	7.31 (0.07 to 7.54)
42	1.22 (0.01 to 1.25)	1.33 (0.01 to 1.37)	7.02 (0.07 to 7.23)	7.62 (0.07 to 7.88)
43	1.27 (0.01 to 1.31)	1.38 (0.01 to 1.44)	7.32 (0.07 to 7.55)	7.94 (0.08 to 8.22)
44	1.33 (0.01 to 1.37)	1.44 (0.01 to 1.50)	7.62 (0.07 to 7.89)	8.27 (0.08 to 8.59)
45	1.38 (0.01 to 1.43)	1.51 (0.01 to 1.57)	7.94 (0.08 to 8.25)	8.61 (0.08 to 8.97)
46	1.44 (0.01 to 1.50)	1.57 (0.01 to 1.64)	8.27 (0.08 to 8.62)	8.97 (0.09 to 9.37)
47	1.51 (0.01 to 1.57)	1.64 (0.02 to 1.72)	8.61 (0.08 to 9.01)	9.34 (0.09 to 9.79)
48	1.57 (0.01 to 1.65)	1.71 (0.02 to 1.80)	8.97 (0.09 to 9.41)	9.73 (0.09 to 10.23)
49	1.64 (0.02 to 1.72)	1.78 (0.02 to 1.89)	9.34 (0.09 to 9.83)	10.13 (0.10 to 10.68)
**Low birth weight**				
Maternal age (years)	nIVF_S	IVF_S	nIVF_T	IVF_T
20	5.89 (0.06 to 5.96)	6.45 (0.06 to 6.55)	38.62 (0.38 to 39.04)	41.52 (0.41 to 42.02)
21	5.44 (0.05 to 5.49)	5.96 (0.06 to 6.05)	36.24 (0.36 to 36.56)	39.02 (0.39 to 39.43)
22	5.03 (0.05 to 5.07)	5.52 (0.05 to 5.58)	33.98 (0.34 to 34.25)	36.64 (0.36 to 37.00)
23	4.67 (0.05 to 4.70)	5.12 (0.05 to 5.18)	31.91 (0.32 to 32.17)	34.46 (0.34 to 34.80)
24	4.36 (0.04 to 4.39)	4.78 (0.05 to 4.84)	30.13 (0.30 to 30.37)	32.57 (0.32 to 32.90)
25	4.12 (0.04 to 4.14)	4.51 (0.04 to 4.57)	28.68 (0.28 to 28.91)	31.03 (0.31 to 31.34)
26	3.94 (0.04 to 3.96)	4.32 (0.04 to 4.37)	27.63 (0.27 to 27.84)	29.91 (0.30 to 30.20)
27	3.84 (0.04 to 3.87)	4.21 (0.04 to 4.27)	27.02 (0.27 to 27.24)	29.27 (0.29 to 29.56)
28	3.82 (0.04 to 3.84)	4.19 (0.04 to 4.24)	26.90 (0.27 to 27.10)	29.13 (0.29 to 29.41)
29	3.85 (0.04 to 3.88)	4.23 (0.04 to 4.28)	27.10 (0.27 to 27.32)	29.35 (0.29 to 29.64)
30	3.92 (0.04 to 3.94)	4.30 (0.04 to 4.35)	27.49 (0.27 to 27.70)	29.76 (0.29 to 30.05)
31	4.01 (0.04 to 4.04)	4.40 (0.04 to 4.45)	28.04 (0.28 to 28.27)	30.35 (0.30 to 30.65)
32	4.13 (0.04 to 4.16)	4.53 (0.04 to 4.58)	28.77 (0.29 to 28.99)	31.12 (0.31 to 31.42)
33	4.27 (0.04 to 4.30)	4.68 (0.05 to 4.74)	29.61 (0.29 to 29.83)	32.02 (0.32 to 32.31)
34	4.43 (0.04 to 4.45)	4.86 (0.05 to 4.91)	30.53 (0.30 to 30.76)	32.99 (0.33 to 33.29)
35	4.60 (0.05 to 4.63)	5.04 (0.05 to 5.10)	31.51 (0.31 to 31.75)	34.03 (0.34 to 34.34)
36	4.78 (0.05 to 4.81)	5.24 (0.05 to 5.30)	32.54 (0.32 to 32.79)	35.12 (0.35 to 35.45)
37	4.97 (0.05 to 5.00)	5.45 (0.05 to 5.51)	33.63 (0.33 to 33.89)	36.27 (0.36 to 36.61)
38	5.17 (0.05 to 5.21)	5.67 (0.06 to 5.74)	34.77 (0.35 to 35.03)	37.47 (0.37 to 37.81)
39	5.39 (0.05 to 5.42)	5.91 (0.06 to 5.98)	35.95 (0.36 to 36.23)	38.71 (0.38 to 39.07)

©2021 Wang Y et al. *JAMA Network Open*.

40	5.61 (0.06 to 5.66)	6.15 (0.06 to 6.23)	37.16 (0.37 to 37.47)	39.99 (0.40 to 40.37)
41	5.85 (0.06 to 5.90)	6.41 (0.06 to 6.49)	38.40 (0.38 to 38.75)	41.29 (0.41 to 41.71)
42	6.09 (0.06 to 6.16)	6.67 (0.07 to 6.77)	39.67 (0.39 to 40.07)	42.62 (0.42 to 43.09)
43	6.34 (0.06 to 6.42)	6.95 (0.07 to 7.06)	40.97 (0.41 to 41.42)	43.98 (0.43 to 44.50)
44	6.61 (0.07 to 6.70)	7.24 (0.07 to 7.36)	42.29 (0.42 to 42.81)	45.36 (0.45 to 45.94)
45	6.88 (0.07 to 6.99)	7.54 (0.07 to 7.68)	43.64 (0.43 to 44.23)	46.76 (0.46 to 47.40)
46	7.17 (0.07 to 7.30)	7.85 (0.08 to 8.01)	45.02 (0.44 to 45.67)	48.18 (0.47 to 48.90)
47	7.46 (0.07 to 7.61)	8.17 (0.08 to 8.35)	46.41 (0.46 to 47.15)	49.63 (0.49 to 50.42)
48	7.77 (0.08 to 7.94)	8.51 (0.08 to 8.71)	47.83 (0.47 to 48.64)	51.09 (0.50 to 51.96)
49	8.09 (0.08 to 8.29)	8.86 (0.09 to 9.09)	49.27 (0.48 to 50.16)	52.57 (0.52 to 53.52)
**Very low birth weight**				
Maternal age (years)	nIVF_S	IVF_S	nIVF_T	IVF_T
20	0.82 (0.01 to 0.85)	0.94 (0.01 to 0.98)	3.90 (0.04 to 4.05)	4.44 (0.04 to 4.63)
21	0.79 (0.01 to 0.81)	0.90 (0.01 to 0.94)	3.76 (0.04 to 3.88)	4.28 (0.04 to 4.43)
22	0.76 (0.01 to 0.78)	0.87 (0.01 to 0.90)	3.62 (0.04 to 3.72)	4.12 (0.04 to 4.26)
23	0.74 (0.01 to 0.75)	0.84 (0.01 to 0.87)	3.49 (0.03 to 3.58)	3.97 (0.04 to 4.10)
24	0.71 (0.01 to 0.72)	0.81 (0.01 to 0.83)	3.36 (0.03 to 3.45)	3.82 (0.04 to 3.94)
25	0.68 (0.01 to 0.69)	0.77 (0.01 to 0.80)	3.23 (0.03 to 3.31)	3.67 (0.04 to 3.79)
26	0.65 (0.01 to 0.66)	0.74 (0.01 to 0.77)	3.11 (0.03 to 3.18)	3.53 (0.03 to 3.64)
27	0.64 (0.01 to 0.65)	0.73 (0.01 to 0.75)	3.04 (0.03 to 3.12)	3.46 (0.03 to 3.57)
28	0.64 (0.01 to 0.65)	0.73 (0.01 to 0.76)	3.06 (0.03 to 3.13)	3.48 (0.03 to 3.58)
29	0.66 (0.01 to 0.67)	0.75 (0.01 to 0.77)	3.13 (0.03 to 3.21)	3.56 (0.03 to 3.67)
30	0.68 (0.01 to 0.69)	0.77 (0.01 to 0.80)	3.21 (0.03 to 3.29)	3.65 (0.04 to 3.77)
31	0.70 (0.01 to 0.71)	0.80 (0.01 to 0.82)	3.32 (0.03 to 3.40)	3.78 (0.04 to 3.89)
32	0.73 (0.01 to 0.74)	0.83 (0.01 to 0.86)	3.47 (0.03 to 3.55)	3.94 (0.04 to 4.06)
33	0.77 (0.01 to 0.78)	0.87 (0.01 to 0.90)	3.64 (0.04 to 3.72)	4.14 (0.04 to 4.26)
34	0.80 (0.01 to 0.82)	0.92 (0.01 to 0.94)	3.81 (0.04 to 3.90)	4.33 (0.04 to 4.46)
35	0.84 (0.01 to 0.85)	0.96 (0.01 to 0.99)	3.98 (0.04 to 4.07)	4.52 (0.04 to 4.66)
36	0.87 (0.01 to 0.89)	1.00 (0.01 to 1.03)	4.14 (0.04 to 4.25)	4.71 (0.05 to 4.85)
37	0.91 (0.01 to 0.92)	1.04 (0.01 to 1.07)	4.30 (0.04 to 4.41)	4.89 (0.05 to 5.04)
38	0.95 (0.01 to 0.96)	1.08 (0.01 to 1.11)	4.47 (0.04 to 4.58)	5.08 (0.05 to 5.23)
39	0.98 (0.01 to 1.00)	1.12 (0.01 to 1.15)	4.63 (0.05 to 4.75)	5.27 (0.05 to 5.43)
40	1.02 (0.01 to 1.04)	1.16 (0.01 to 1.20)	4.80 (0.05 to 4.93)	5.46 (0.05 to 5.63)
41	1.06 (0.01 to 1.08)	1.20 (0.01 to 1.25)	4.98 (0.05 to 5.13)	5.65 (0.05 to 5.85)
42	1.09 (0.01 to 1.12)	1.25 (0.01 to 1.29)	5.16 (0.05 to 5.33)	5.86 (0.06 to 6.08)
43	1.14 (0.01 to 1.17)	1.29 (0.01 to 1.35)	5.35 (0.05 to 5.54)	6.07 (0.06 to 6.31)
44	1.18 (0.01 to 1.22)	1.34 (0.01 to 1.40)	5.54 (0.05 to 5.76)	6.29 (0.06 to 6.57)
45	1.22 (0.01 to 1.27)	1.39 (0.01 to 1.46)	5.74 (0.05 to 6.00)	6.52 (0.06 to 6.83)
46	1.27 (0.01 to 1.32)	1.44 (0.01 to 1.52)	5.95 (0.06 to 6.24)	6.75 (0.06 to 7.10)
47	1.31 (0.01 to 1.38)	1.50 (0.01 to 1.58)	6.16 (0.06 to 6.49)	7.00 (0.07 to 7.39)
48	1.36 (0.01 to 1.44)	1.55 (0.01 to 1.65)	6.39 (0.06 to 6.76)	7.25 (0.07 to 7.68)
49	1.41 (0.01 to 1.50)	1.61 (0.02 to 1.72)	6.62 (0.06 to 7.03)	7.51 (0.07 to 7.99)

©2021 Wang Y et al. *JAMA Network Open*.

Macrosomia				
Maternal age (years)	nIVF_S	IVF_S	nIVF_T	IVF_T
20	3.12 (0.03 to 3.18)	3.06 (0.03 to 3.14)	0.17 (0.00 to 0.18)	0.16 (0.00 to 0.17)
21	3.42 (0.03 to 3.46)	3.35 (0.03 to 3.42)	0.18 (0.00 to 0.20)	0.18 (0.00 to 0.19)
22	3.74 (0.04 to 3.77)	3.66 (0.04 to 3.73)	0.20 (0.00 to 0.21)	0.20 (0.00 to 0.21)
23	4.07 (0.04 to 4.10)	3.98 (0.04 to 4.06)	0.22 (0.00 to 0.23)	0.21 (0.00 to 0.23)
24	4.39 (0.04 to 4.42)	4.30 (0.04 to 4.39)	0.24 (0.00 to 0.25)	0.23 (0.00 to 0.25)
25	4.69 (0.05 to 4.72)	4.59 (0.05 to 4.68)	0.25 (0.00 to 0.27)	0.25 (0.00 to 0.26)
26	4.94 (0.05 to 4.97)	4.84 (0.05 to 4.93)	0.27 (0.00 to 0.28)	0.26 (0.00 to 0.28)
27	5.15 (0.05 to 5.19)	5.05 (0.05 to 5.15)	0.28 (0.00 to 0.30)	0.27 (0.00 to 0.29)
28	5.36 (0.05 to 5.39)	5.25 (0.05 to 5.35)	0.29 (0.00 to 0.31)	0.28 (0.00 to 0.30)
29	5.53 (0.05 to 5.56)	5.41 (0.05 to 5.52)	0.30 (0.00 to 0.32)	0.29 (0.00 to 0.31)
30	5.62 (0.06 to 5.66)	5.51 (0.05 to 5.62)	0.31 (0.00 to 0.32)	0.30 (0.00 to 0.32)
31	5.72 (0.06 to 5.75)	5.60 (0.05 to 5.71)	0.31 (0.00 to 0.33)	0.30 (0.00 to 0.32)
32	5.86 (0.06 to 5.90)	5.74 (0.06 to 5.85)	0.32 (0.00 to 0.34)	0.31 (0.00 to 0.33)
33	6.02 (0.06 to 6.05)	5.90 (0.06 to 6.01)	0.33 (0.00 to 0.35)	0.32 (0.00 to 0.34)
34	6.14 (0.06 to 6.18)	6.02 (0.06 to 6.13)	0.33 (0.00 to 0.36)	0.33 (0.00 to 0.35)
35	6.22 (0.06 to 6.26)	6.09 (0.06 to 6.21)	0.34 (0.00 to 0.36)	0.33 (0.00 to 0.35)
36	6.26 (0.06 to 6.30)	6.13 (0.06 to 6.25)	0.34 (0.00 to 0.36)	0.33 (0.00 to 0.36)
37	6.26 (0.06 to 6.30)	6.14 (0.06 to 6.26)	0.34 (0.00 to 0.36)	0.33 (0.00 to 0.36)
38	6.25 (0.06 to 6.29)	6.12 (0.06 to 6.24)	0.34 (0.00 to 0.36)	0.33 (0.00 to 0.35)
39	6.22 (0.06 to 6.26)	6.09 (0.06 to 6.21)	0.34 (0.00 to 0.36)	0.33 (0.00 to 0.35)
40	6.18 (0.06 to 6.23)	6.06 (0.06 to 6.18)	0.34 (0.00 to 0.36)	0.33 (0.00 to 0.35)
41	6.14 (0.06 to 6.20)	6.02 (0.06 to 6.14)	0.33 (0.00 to 0.36)	0.33 (0.00 to 0.35)
42	6.10 (0.06 to 6.18)	5.98 (0.06 to 6.11)	0.33 (0.00 to 0.35)	0.33 (0.00 to 0.35)
43	6.07 (0.06 to 6.15)	5.95 (0.06 to 6.08)	0.33 (0.00 to 0.35)	0.32 (0.00 to 0.34)
44	6.03 (0.06 to 6.12)	5.91 (0.06 to 6.05)	0.33 (0.00 to 0.35)	0.32 (0.00 to 0.34)
45	5.99 (0.06 to 6.10)	5.87 (0.06 to 6.02)	0.33 (0.00 to 0.35)	0.32 (0.00 to 0.34)
46	5.95 (0.06 to 6.07)	5.83 (0.06 to 5.99)	0.32 (0.00 to 0.35)	0.32 (0.00 to 0.34)
47	5.92 (0.06 to 6.05)	5.80 (0.06 to 5.97)	0.32 (0.00 to 0.34)	0.31 (0.00 to 0.34)
48	5.88 (0.06 to 6.03)	5.76 (0.06 to 5.94)	0.32 (0.00 to 0.34)	0.31 (0.00 to 0.33)
49	5.84 (0.06 to 6.00)	5.73 (0.06 to 5.91)	0.32 (0.00 to 0.34)	0.31 (0.00 to 0.33)
**Malformation**				
Maternal age (years)	nIVF_S	IVF_S	nIVF_T	IVF_T
20	0.95 (0.01 to 0.98)	1.04 (0.01 to 1.09)	2.87 (0.03 to 2.98)	3.13 (0.03 to 3.27)
21	0.97 (0.01 to 0.99)	1.06 (0.01 to 1.10)	2.92 (0.03 to 3.01)	3.19 (0.03 to 3.30)
22	0.99 (0.01 to 1.00)	1.08 (0.01 to 1.11)	2.97 (0.03 to 3.04)	3.24 (0.03 to 3.35)
23	1.01 (0.01 to 1.02)	1.10 (0.01 to 1.14)	3.03 (0.03 to 3.10)	3.31 (0.03 to 3.41)
24	1.03 (0.01 to 1.05)	1.13 (0.01 to 1.17)	3.11 (0.03 to 3.19)	3.40 (0.03 to 3.51)
25	1.07 (0.01 to 1.09)	1.17 (0.01 to 1.21)	3.23 (0.03 to 3.31)	3.53 (0.03 to 3.64)
26	1.13 (0.01 to 1.14)	1.23 (0.01 to 1.27)	3.38 (0.03 to 3.46)	3.70 (0.04 to 3.81)

©2021 Wang Y et al. *JAMA Network Open*.

27	1.17 (0.01 to 1.19)	1.28 (0.01 to 1.32)	3.52 (0.03 to 3.60)	3.84 (0.04 to 3.96)
28	1.19 (0.01 to 1.20)	1.30 (0.01 to 1.34)	3.57 (0.03 to 3.65)	3.90 (0.04 to 4.01)
29	1.20 (0.01 to 1.22)	1.32 (0.01 to 1.36)	3.61 (0.04 to 3.70)	3.94 (0.04 to 4.06)
30	1.24 (0.01 to 1.25)	1.35 (0.01 to 1.40)	3.72 (0.04 to 3.80)	4.06 (0.04 to 4.18)
31	1.28 (0.01 to 1.30)	1.40 (0.01 to 1.44)	3.83 (0.04 to 3.93)	4.19 (0.04 to 4.31)
32	1.30 (0.01 to 1.32)	1.42 (0.01 to 1.47)	3.90 (0.04 to 4.00)	4.26 (0.04 to 4.39)
33	1.31 (0.01 to 1.33)	1.44 (0.01 to 1.48)	3.94 (0.04 to 4.03)	4.30 (0.04 to 4.43)
34	1.33 (0.01 to 1.34)	1.45 (0.01 to 1.50)	3.98 (0.04 to 4.07)	4.34 (0.04 to 4.47)
35	1.34 (0.01 to 1.36)	1.47 (0.01 to 1.51)	4.02 (0.04 to 4.12)	4.39 (0.04 to 4.53)
36	1.36 (0.01 to 1.38)	1.49 (0.01 to 1.53)	4.08 (0.04 to 4.18)	4.45 (0.04 to 4.58)
37	1.38 (0.01 to 1.40)	1.51 (0.01 to 1.56)	4.14 (0.04 to 4.24)	4.52 (0.04 to 4.65)
38	1.40 (0.01 to 1.42)	1.53 (0.01 to 1.58)	4.20 (0.04 to 4.30)	4.58 (0.04 to 4.72)
39	1.42 (0.01 to 1.45)	1.56 (0.02 to 1.61)	4.26 (0.04 to 4.37)	4.66 (0.05 to 4.80)
40	1.45 (0.01 to 1.47)	1.58 (0.02 to 1.63)	4.33 (0.04 to 4.45)	4.73 (0.05 to 4.88)
41	1.47 (0.01 to 1.50)	1.61 (0.02 to 1.66)	4.40 (0.04 to 4.53)	4.81 (0.05 to 4.97)
42	1.50 (0.01 to 1.53)	1.63 (0.02 to 1.70)	4.47 (0.04 to 4.62)	4.89 (0.05 to 5.06)
43	1.52 (0.01 to 1.56)	1.66 (0.02 to 1.73)	4.55 (0.04 to 4.71)	4.96 (0.05 to 5.16)
44	1.54 (0.01 to 1.60)	1.69 (0.02 to 1.76)	4.62 (0.04 to 4.80)	5.04 (0.05 to 5.26)
45	1.57 (0.02 to 1.63)	1.72 (0.02 to 1.80)	4.70 (0.05 to 4.90)	5.13 (0.05 to 5.36)
46	1.60 (0.02 to 1.66)	1.74 (0.02 to 1.83)	4.77 (0.05 to 4.99)	5.21 (0.05 to 5.47)
47	1.62 (0.02 to 1.70)	1.77 (0.02 to 1.87)	4.85 (0.05 to 5.10)	5.29 (0.05 to 5.58)
48	1.65 (0.02 to 1.74)	1.80 (0.02 to 1.91)	4.93 (0.05 to 5.20)	5.38 (0.05 to 5.69)
49	1.68 (0.02 to 1.77)	1.83 (0.02 to 1.95)	5.01 (0.05 to 5.31)	5.46 (0.05 to 5.80)
**Stillbirth**				
Maternal age (years)	nIVF_S	IVF_S	nIVF_T	IVF_T
20	0.45 (0.00 to 0.47)	0.30 (0.00 to 0.32)	4.30 (0.04 to 4.50)	2.93 (0.03 to 3.10)
21	0.40 (0.00 to 0.41)	0.27 (0.00 to 0.28)	3.82 (0.04 to 3.97)	2.60 (0.02 to 2.73)
22	0.35 (0.00 to 0.36)	0.24 (0.00 to 0.25)	3.40 (0.03 to 3.51)	2.31 (0.02 to 2.42)
23	0.32 (0.00 to 0.32)	0.21 (0.00 to 0.22)	3.05 (0.03 to 3.14)	2.07 (0.02 to 2.17)
24	0.29 (0.00 to 0.29)	0.19 (0.00 to 0.20)	2.77 (0.03 to 2.86)	1.88 (0.02 to 1.97)
25	0.27 (0.00 to 0.27)	0.18 (0.00 to 0.19)	2.58 (0.03 to 2.66)	1.75 (0.02 to 1.84)
26	0.26 (0.00 to 0.26)	0.17 (0.00 to 0.18)	2.48 (0.02 to 2.55)	1.68 (0.02 to 1.76)
27	0.25 (0.00 to 0.26)	0.17 (0.00 to 0.18)	2.41 (0.02 to 2.49)	1.64 (0.02 to 1.72)
28	0.25 (0.00 to 0.25)	0.17 (0.00 to 0.17)	2.38 (0.02 to 2.45)	1.61 (0.02 to 1.69)
29	0.25 (0.00 to 0.25)	0.17 (0.00 to 0.18)	2.40 (0.02 to 2.47)	1.63 (0.02 to 1.70)
30	0.26 (0.00 to 0.27)	0.18 (0.00 to 0.18)	2.51 (0.02 to 2.58)	1.70 (0.02 to 1.78)
31	0.28 (0.00 to 0.28)	0.19 (0.00 to 0.20)	2.67 (0.03 to 2.75)	1.81 (0.02 to 1.89)
32	0.29 (0.00 to 0.30)	0.20 (0.00 to 0.21)	2.81 (0.03 to 2.89)	1.91 (0.02 to 1.99)
33	0.31 (0.00 to 0.31)	0.21 (0.00 to 0.22)	2.94 (0.03 to 3.03)	2.00 (0.02 to 2.09)
34	0.32 (0.00 to 0.33)	0.22 (0.00 to 0.23)	3.11 (0.03 to 3.20)	2.12 (0.02 to 2.21)
35	0.34 (0.00 to 0.35)	0.23 (0.00 to 0.24)	3.32 (0.03 to 3.42)	2.26 (0.02 to 2.36)
36	0.37 (0.00 to 0.38)	0.25 (0.00 to 0.26)	3.56 (0.03 to 3.67)	2.42 (0.02 to 2.53)

©2021 Wang Y et al. *JAMA Network Open*.

37	0.40 (0.00 to 0.41)	0.27 (0.00 to 0.28)	3.85 (0.04 to 3.97)	2.62 (0.03 to 2.74)
38	0.44 (0.00 to 0.45)	0.29 (0.00 to 0.31)	4.17 (0.04 to 4.30)	2.84 (0.03 to 2.97)
39	0.47 (0.00 to 0.49)	0.32 (0.00 to 0.34)	4.54 (0.04 to 4.68)	3.09 (0.03 to 3.23)
40	0.52 (0.01 to 0.53)	0.35 (0.00 to 0.37)	4.95 (0.05 to 5.11)	3.37 (0.03 to 3.53)
41	0.56 (0.01 to 0.58)	0.38 (0.00 to 0.40)	5.39 (0.05 to 5.58)	3.68 (0.04 to 3.85)
42	0.62 (0.01 to 0.64)	0.42 (0.00 to 0.44)	5.86 (0.06 to 6.09)	4.01 (0.04 to 4.21)
43	0.67 (0.01 to 0.70)	0.46 (0.00 to 0.48)	6.38 (0.06 to 6.66)	4.36 (0.04 to 4.60)
44	0.73 (0.01 to 0.77)	0.50 (0.00 to 0.53)	6.95 (0.07 to 7.28)	4.75 (0.04 to 5.03)
45	0.80 (0.01 to 0.84)	0.54 (0.01 to 0.58)	7.56 (0.07 to 7.95)	5.18 (0.05 to 5.50)
46	0.87 (0.01 to 0.92)	0.59 (0.01 to 0.63)	8.22 (0.08 to 8.69)	5.64 (0.05 to 6.01)
47	0.95 (0.01 to 1.01)	0.65 (0.01 to 0.69)	8.94 (0.08 to 9.50)	6.14 (0.06 to 6.57)
48	1.04 (0.01 to 1.11)	0.71 (0.01 to 0.76)	9.72 (0.09 to 10.38)	6.68 (0.06 to 7.19)
49	1.14 (0.01 to 1.22)	0.77 (0.01 to 0.83)	10.56 (0.10 to 11.33)	7.27 (0.07 to 7.86)

nIVF_S: singleton pregnancy conceived with non-IVF; nIVF_T: twin pregnancy conceived with non-IVF; IVF_S: singleton pregnancy conceived with IVF; IVF_T: twin pregnancy conceived with IVF.

©2021 Wang Y et al. *JAMA Network Open*.
